# A Spontaneous Complementary Mutation Restores the RNA Silencing Suppression Activity of HC-Pro and the Virulence of Sugarcane Mosaic Virus

**DOI:** 10.3389/fpls.2020.01279

**Published:** 2020-08-21

**Authors:** Xiao-Jie Xu, Huan-Gai Li, De-Jie Cheng, Ling-Zhi Liu, Chao Geng, Yan-Ping Tian, Xiang-Dong Li

**Affiliations:** ^1^ Shandong Province Key Laboratory for Agricultural Microbiology, Laboratory of Plant Virology, Department of Plant Pathology, College of Plant Protection, Shandong Agricultural University, Tai’an, China; ^2^ Protein Science Laboratory of Ministry of Education, School of Life Sciences, Tsinghua University, Beijing, China

**Keywords:** helper component-proteinase, *Potyvirus*, RNA silencing suppression, spontaneous mutation, sugarcane mosaic virus, virulence

## Abstract

Cross-protection is a promising measure to control plant viral diseases. Reverse genetics had been recently adopted to generate attenuated mutants that have potential in cross-protection. But studies on the variability of the progeny viruses of the attenuated mutants are scarce. Sugarcane mosaic virus (SCMV; genus *Potyvirus*, family *Potyviridae*) is the prevalent virus inducing maize dwarf mosaic disease in China. Here, we showed that the substitution of arginine with isoleucine in the FRNK motif at position 184 of helper component-proteinase (HC-Pro) abolished its RNA silencing suppression (RSS) activity, drastically reduced the virulence and accumulation level of SCMV, and impaired the synergism between SCMV and maize chlorotic mottle virus. The attenuated mutant could protect maize plants from a severe infection of SCMV. However, a spontaneous mutation of glycine at position 440 to arginine in HC-Pro rescued the virulence and synergism with maize chlorotic mottle virus of SCMV and the RSS activity of HC-Pro. Similar results were obtained with tobacco vein banding mosaic virus and watermelon mosaic virus. These results provide novel evidence for the complementary mutation of potyviruses in maintaining the HC-Pro RSS activity and potyviral virulence and remind us of evaluating the potential risk of attenuated mutants thoroughly before applying for the control of plant viral diseases *via* cross-protection.

## Introduction

Cross-protection is the phenomenon that plants infected with one mild strain of a virus will escape or prevent the infection by closely related severe viruses (Kunkel, 1955). It has been used for the control of more than 30 viruses including citrus tristeza virus (CTV), papaya ringspot virus (PRSV), potato virus X (PVX), potato virus Y (PVY), sugarcane mosaic virus (SCMV), and tobacco mosaic virus (TMV) in the glasshouses or the fields (Rast, 1972; Krstic et al., 1995; You et al., 2005; Folimonova, 2013; Cong et al., 2019; Cheng et al., 2020). Cross-protection is an environmentally safe and friendly disease management strategy and meets the societal demand for high-quality and sustainable agricultural production systems (Pechinger et al., 2019).

The availability and safety of mild strains (or attenuated mutants) are two limiting factors affecting the application of cross-protection in practice. The mild strains for cross-protection can be obtained from naturally occurring strains with slight or no distinct symptoms, and attenuated mutants can be obtained from the treatment with mutagenic agents or heat (Tomlinson and Shepherd, 1978; Tanzi et al., 1987; Chiang et al., 2007). Currently, potential attenuated mutants can also be obtained using reverse genetics and the use of high-throughput sequencing techniques (Lin et al., 2007; Cook et al., 2016; Kamitani et al., 2016; Huang et al., 2019; Tuo et al., 2020). With advances in biotechnologies and bioinformatics tools, the attainment of attenuated mutants of pathogenic viruses is now more affordable so that the application of cross-protection is expected to increase (Pechinger et al., 2019). The safety of mild strains or attenuated mutants is the primary consideration in the application of cross-protection (Ziebell and MacDiarmid, 2017). Reversal mutation and synergism are two of the biosafety factors limiting the application of cross-protection. The attenuated mutants are at risk of mutation into virulent ones when used for viral disease control. Reversal mutation of attenuated mutants to wild type ones is frequently reported. After five passages, reversal mutation of FINK to the wild type motif FRNK in TEV HC-Pro was detected in two of two lineages tested (Ambrós et al., 2018). Synergism is the phenomenon that the infection of plants by two unrelated viruses results in a remarkable increase in both symptom expression and virus accumulation beyond the single infection (Bance, 1991; Yang and Ravelonandro, 2002; Gonzalez-Jara et al., 2005). The potential mild strains or attenuated mutants should have no synergism with other viruses infecting the target host plants. However, studies on the safety of progeny viruses of the attenuated mutants are scarce.

The genus *Potyvirus* (family *Potyviridae*) is the largest group of plant-infecting RNA viruses. The potyviral genome is about 10,000 nucleotides (nt) in length and contains two open reading frames (ORFs) encoding two polyproteins that are cleaved into 11 mature proteins (Chung et al., 2008; Olspert et al., 2015). Helper component-proteinase (HC-Pro) is one of the best-studied multifunctional proteins for potyviruses. Besides its self-cleavage from polyprotein, HC-Pro is also involved in aphid transmission, movement, replication, RNA silencing suppression (RSS), virulence, the final yield of viruses, and synergism with viruses from other genera (Yang and Ravelonandro, 2002; Valli et al., 2014; Ivanov et al., 2016; Poque et al., 2017; Valli et al., 2018). Some nonsynonymous mutants in HC-Pro resulted in attenuated virulence in some potyviruses. A mutation of aspartic acid (D) to tyrosine (Y) at position 193 in HC-Pro drastically reduced the symptoms caused by clover yellow vein virus (ClYVV) in legume plants (Yambao et al., 2008). The mutation of lycine (K) at position 125 to D or glycine (G) at position 317 to K in HC-Pro significantly reduced the virulence of PRSV (Huang et al., 2019). The role of the conserved FRNK motif of HC-Pro in potyvirus virulence has been extensively investigated. However, the results are inconsistent for different potyviruses. The mutation of arginine (R) to isoleucine (I) in FRNK motif of HC-Pro significantly reduced the virulence of tobacco vein banding mosaic virus (TVBMV) and zucchini yellow mosaic virus (ZYMV), while the FINK mutants of tobacco etch virus (TEV) and turnip mosaic virus (TuMV) both lost their infectivity (Shiboleth et al., 2007; Gao et al., 2012). The FKNK mutant of TuMV caused mild mottling symptoms in the inoculated leaves and no symptoms in the upper non-inoculated leaves of *Nicotiana benthamiana* plants; however, the FKNK mutant of ZYMV caused severe squash leaf symptoms (Shiboleth et al., 2007; Kung et al., 2014). The HC-Pro is also implicated in the synergism of potyviruses with other viruses. In the synergistic interaction of plum pox virus (PPV) and PVX, the leucine (L) at position 134 and K in PTK motif of PPV HC-Pro is the amino acid determinants (Yang and Ravelonandro, 2002; Gonzalez-Jara et al., 2005).

Maize dwarf mosaic (MDM), which was first reported in Ohio of the USA in 1963 (Janson and Ellett, 1963), is one of the most widespread viral diseases of maize in the world (Wu et al., 2012; Moradi et al., 2017). MDM was first observed in the Henan province of China in 1968 and has spread to all the maize-growing regions in China (Gao et al., 2011; Xie et al., 2016). SCMV is the most prevalent virus inducing MDM in China and causes yield losses of 10–60% (Jiang and Zhou, 2002; Yan et al., 2016).

The synergistic infection of maize plants with maize chlorotic mottle virus (MCMV), a plus-sense ssRNA virus of the genus *Machlomovirus*, family *Tombusviridae*, and SCMV induces severe mosaic and lethal necrosis and results in a dramatic increase in the accumulation levels of MCMV (Xia et al., 2016). So far, there has been no report on the critical amino acids regulating the virulence of SCMV, or the amino acid determinants of SCMV for MCMV–SCMV synergism.

In this study, by using site-directed mutagenesis, we introduced mutations to the HC-Pro gene and obtained attenuated SCMV mutants that could protect maize plants from severe infection. However, the mutant could restore virulence by a spontaneous mutation, which also restored the RSS activity of HC-Pro and synergism of SCMV and MCMV. These results enhance our understanding of potyviruses pathogenesis and remind us of the importance of assessing and reducing the risks involved in the use of attenuated mutants in cross-protection application.

## Materials and Methods

### Plant Growth and Virus Inoculation

Maize (*Zea mays*) inbred line B73, *Nicotiana benthamiana*, GFP-expressing *N. benthamiana* (16C), and *Cucumis melo* plants were cultivated in a growth chamber with 16 h light (24°C) and 8 h dark (22°C) cycles.

All plasmids were introduced individually into *Agrobacterium* strain GV3101 by the freeze/thaw method (Hofgen and Willmitzer, 1988). The transformed *Agrobacterium* cultures were grown overnight in the Luria–Bertani culture medium containing 50 µg/ml kanamycin and 50 µg/ml rifampicin followed by 3 h incubation in an induction buffer [10 mM MgCl_2_, 150 μM acetosyringone and 10 mM 2-(N-Morpholino) ethane sulfonic acid (MES)] at room temperature. Individual *Agrobacterium* cultures were adjusted to OD_600_ = 0.5 for virus inoculation (Geng et al., 2017). The diluted *Agrobacterium* cultures of watermelon mosaic virus (WMV)-based and TVBMV-based constructs were infiltrated individually into leaves of *Cucumis melo* and *N*. *benthamiana* plants using needleless syringes. For SCMV inoculation, the diluted *Agrobacterium* cultures of SCMV-based constructs were mixed with *Agrobacterium* cells harboring tomato bushy stunt virus (TBSV) p19-expressing binary plasmid pBin-p19 and infiltrated into the leaves of *N. benthamiana* plants. The infiltrated leaves were harvested after three days post infiltration, ground in 20 mM phosphate-buffered saline (pH 7.2), and centrifuged at 3,000 *g* for 3 min. The supernatants were mechanically inoculated onto leaves of the two-leaf staged maize plants. Each experiment was repeated independently for three times, with ten plants for each viral construct in each experiment. Crude extracts from SCMV or MCMV-infected maize leaves, ground in 20 mM phosphate-buffered saline (pH 7.2), were equally mixed as the source of co-infection.

### Plasmid Construction

The infectious clone of SCMV was kindly provided by Professor Yule Liu from Tsinghua University, China. The GFP-expressing infectious clones of TVBMV and WMV were constructed and modified in the Laboratory of Plant Virology, Shandong Agricultural University (Gao et al., 2012; Geng et al., 2017; and unpublished data). Site-directed mutagenesis was performed through PCR using specific primers (**Supplemental Table 1**). For transient expression experiments, DNA fragments were ligated into the binary vector pBin121 between *Bam* HI and *Sac* I sites.

### Cross-Protection Test

For protective inoculation, maize seedlings at the two-leaf stage were mechanically inoculated with crude extracts of *N. benthamiana* leaves obtained three days after infiltration with *Agrobacterium* cultures harboring pBin-p19 and SCMV-HC_FINK_ mutant without the *gfp* gene (ground in 20 mM phosphate-buffered saline, pH 7.2). At five, seven, and ten days, the first fully expanded maize leaves were mechanically challenged with extracts of maize plants infected with SCMV with *gfp* reporter gene (SCMV-GFP), prepared from 0.5 g infected maize leaves in 5 ml 20 mM phosphate-buffered saline (pH 7.2). Protection was evaluated by symptom development and GFP fluorescence on the plants. The accumulation levels of the SCMV were determined by western blotting using a primary antibody against GFP at ten days after the challenge inoculation. Each experiment was repeated three times independently. Maize plants primarily inoculated with empty vector pCB301-Rz were used as a non-protected control.

### RNA Silencing Suppression Assay

Plasmid pBin-GFP and the plasmids expressing wild type and mutant HC-Pros of SCMV, TVBMV, and WMV were individually introduced into competent cells of *Agrobacterium* strain GV3101. The transformed *Agrobacterium* cultures were grown as described above. An equal volume of each *Agrobacterium* culture (OD_600_ = 0.3) was mixed before co-infiltration into *N. benthamiana* 16C leaves. Green ﬂuorescence was observed under UV light and photographed using a Canon 80D camera. The experiments were repeated three times independently.

### RNA Extraction and Quantitative Real-Time Reverse Transcription PCR (qRT-PCR)

Total RNA was extracted from leaf tissues of maize, *N. benthaniana*, 16C, and *Cucumis melo* plants using the Transzol reagent (TransGen Biotech, Beijing, China) and then treated with a gDNA wipe enzyme (Vazyme, Nanjing, China) to eliminate the genomic DNA. The first-strand cDNA for RT-PCR was synthesized from 500 ng total RNA by HiScript^®^ II Q RT SuperMix kit (Vazyme, Nanjing, China) following the manufacturer’s instructions.

The qRT-PCR was performed using ChamQ SYBR qPCR Master Mix (Vazyme, Nanjing, China) on a PCR machine (LC96, Roche, Basel, Switzerland). Gene-specific primers (**Supplemental Table S1**) were designed to amplify housekeeping genes of maize *ZmUbi* gene (GenBank accession: **XM_008647047**), *N. benthamiana* *actin* gene (GenBank accession: **AY179605**), and *Cucumis melo* *EF1α* gene (GenBank accession: **XM_008459007**). The accumulation levels of these genes are stable during viral infection and are therefore used as internal controls for qRT-PCR (Gao et al., 2012; Sun et al., 2014; Chen et al., 2017). Each qRT-PCR was performed with three biological replicates.

### Western Blotting

Preparation of protein samples, SDS-PAGE, electroblotting, and immunodetection were conducted as described previously (Sun and Suzuki, 2008). The antisera against SCMV CP, SCMV HC-Pro, WMV CP, TVBMV CP, and GFP were prepared in the Laboratory of Plant Virology, Shandong Agricultural University (Lan et al., 2007; Wang et al., 2014; Ji et al., 2018; Xu et al., 2018; Xu et al., 2019). The alkaline phosphatase-conjugated goat anti-rabbit immunoglobulin G (IgG) was purchased from Sigma**-**Aldrich, St. Louis, MO, USA. The samples from three biological replicates were detected separately.

### Enzyme-Linked Immunosorbent Assay (ELISA)

The microplate wells were coated with crude extracts from upper non-inoculated leaves of maize, *N benthaniana*, and *C. melo* plants in coating buffer (15 mM Na_2_CO_3_ and 35 mM NaHCO_3_, pH 9.6) and incubated at 37°C for 4 h. The primary antibodies were rabbit polyclonal antibodies against GFP and CPs of SCMV, WMV, and TVBMV, respectively. Alkaline phosphatase-conjugated goat anti-rabbit IgG (1:50,000, v/v) was used as the secondary antibody. After adding a p-Nitrophenyl phosphate substrate solution (Sigma, 0.25 mg/ml), the absorbance value at 405 nm of each well was measured using a Multi-function Microplate Reader (BioTek Synergy™ Mx, Winooski, VT, USA). The ELISA was performed with three biological replicates and three technical replicates.

## Results

### Mutations on the FRNK Motif Reduced the Virulence of SCMV and RSS Activity of HC-Pro

**Figure 1:**
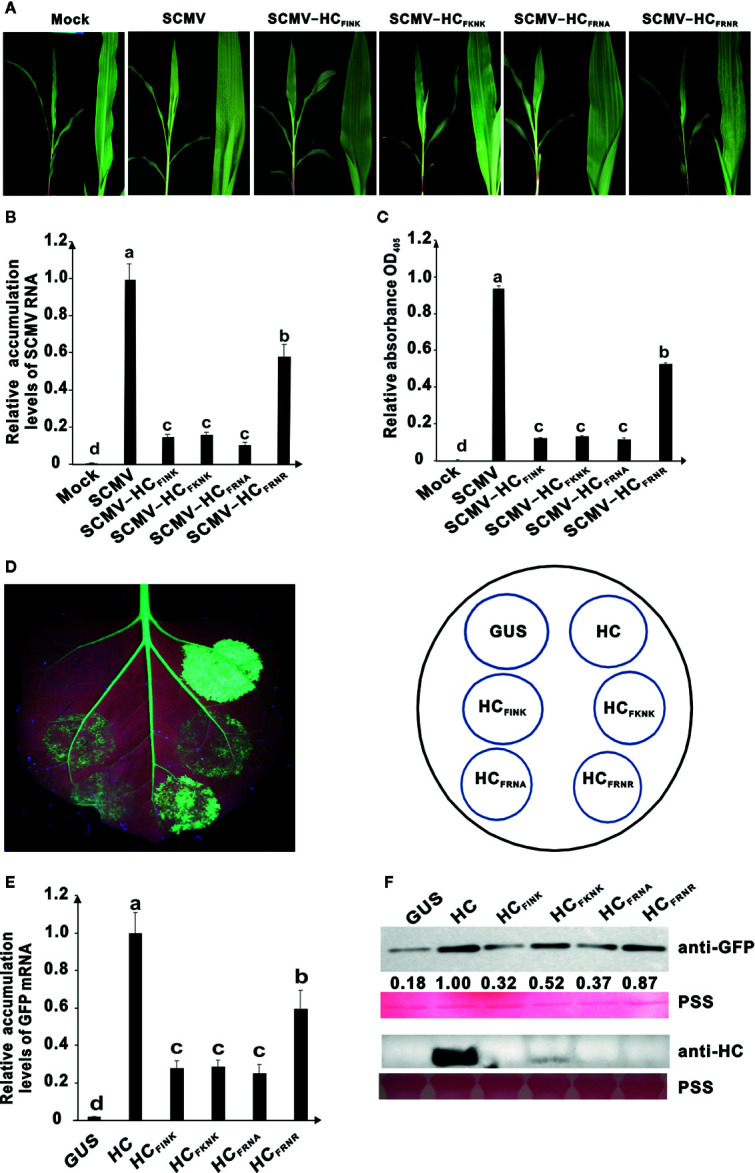
Effects of amino acid mutations in the FRNK motif on virulence, accumulation levels of SCMV, and RNA silencing suppression (RSS) activity of HC-Pro. **(A)** Symptoms caused by wild type and mutant SCMV in maize plants at ten days post inoculation (dpi). The FRNK motif in wild type SCMV HC-Pro was mutated to FINK, FKNK, FRNA, and FRNR in HC-Pros of SCMV-HC_FINK_, SCMV-HC_FKNK_, SCMV-HC_FRNA_, and SCMV-HC_FRNR,_ respectively. Mock, empty vector pCB301-Rz as a negative control. SCMV, wild type SCMV. **(B)** The relative accumulation levels of wild type and mutant SCMV RNA in upper non-inoculated maize leaves at ten dpi. **(C)** The accumulation levels of wild type and mutant SCMV in upper non-inoculated maize leaves at ten dpi. **(D)** RSS activity of wild type and mutants HC-Pro in *Agrobacterium* co-infiltration assay. The GFP-expressing *N. benthamiana* (16C) leaves were co-infiltrated with mixtures of *Agrobacterium* cultures harboring pBin-GFP and either wild type or mutant HC-Pro. The FRNK motif in wild type HC-Pro (HC) was FINK, FKNK, FRNA, and FRNR in HC_FINK_, HC_FKNK_, HC_FRNA_, and HC_FRNR,_ respectively. The GUS was used as a negative control. GFP fluorescence was visualized under long-wavelength UV light and photographed at three days post infiltration. **(E)** The GFP mRNA accumulation levels in agroinfiltrated 16C leaf patches at 3 dpai. **(F)** Western blotting analysis of GFP and HC-Pro accumulation levels in agroinfiltrated leaf patches of 16C at 3 dpai. Lower panel, Ponceau S staining (PSS) as a loading control. The experiments were repeated three times independently. The bar graphs represent the means ± standard deviations of three replicates. Statistically significant differences between means were determined by employing Tukey multiple range test for between-group comparisons. Different letters indicate significant differences (*P* < 0.05). The same below.

The conserved FRNK motif in HC-Pro was reported to be involved in virulence of several potyviruses infecting dicotyledon crops. We conducted site-directed mutagenesis using primers listed in **Supplemental Table 1** and explored the role of R and K at positions 184 and 186 (R^184^ and K^186^), respectively, of the HC-Pro FRNK motif in virulence of SCMV. The resulting plasmids pSCMV-HC_FINK_, pSCMV-HC_FKNK_, pSCMV-HC_FRNA_, and pSCMV-HC_FRNR_ were agroinfiltrated to *Nicotiana benthamiana* leaves separately and mechanically inoculated to maize plants three days later. Three plants were inoculated for each treatment. The corresponding amino acid of R^184^ was mutated to I or K and that of K^186^ was mutated to alanine (A) or R in HC-Pro derived from the progeny of SCMV mutants (**Supplemental Figure 1**). At ten days post inoculation (dpi), wild type SCMV caused severe mosaic and yellowing in the upper non-inoculated leaves of maize, and SCMV-HC_FRNR_ caused less severe mosaic symptoms, but the symptoms caused by SCMV-HC_FINK_, SCMV-HC_FKNK_, and SCMV-HC_FRNA_ were attenuated significantly (**Figure 1A**). Compared with the wild type SCMV, the accumulation levels of viral RNA decreased by about 40% for SCMV-HC_FRNR_ and about 80% for SCMV-HC_FINK_, SCMV-HC_FKNK_, and SCMV-HC_FRNA_ (**Figure 1B**). ELISA results showed that the accumulation levels of CP were consistent with those of SCMV RNA (**Figure 1C**). These results indicated that R^184^ and K^186^ of the FRNK motif were involved in the virulence and accumulation levels of SCMV in maize plants.

Then we investigated whether R^184^ and K^186^ of the FRNK motif are involved in the RSS activity of SCMV HC-Pro. The HC-Pro coding sequence of SCMV was cloned into binary vector pBin121, producing plasmid pBin-HC. Site-directed mutagenesis was conducted using primers listed in **Supplemental Table 1** and produced plasmids pBin-HC_FINK_, pBin-HC_FKNK_, pBin-HC_FRNA_, and pBin-HC_FRNR_. The plasmids were transformed into *Agrobacterium* cells and then mixed with *Agrobacterium* harboring a GFP-expressing binary plasmid pBin-GFP and infiltrated into the leaves of 16C plants. The GUS-expressing plasmid pBin-GUS was used as a negative control. At three days post agroinfiltration (dpai), compared with wild type HC-Pro, the green fluorescence in the 16C leaf patches expressing HC_FRNR_ was weaker and that in the 16C leaf patches expressing HC_FINK_, HC_FKNK_, and HC_FRNA_ was the weakest ones (**Figure 1D**). Results of qRT-PCR showed that GFP mRNA accumulated up to 80% of wild type HC-Pro for HC_FRNR_ and about 30% for HC_FINK_, HC_FKNK_, and HC_FRNA_ (**Figure 1E**). Results of western blotting showed that the GFP accumulation levels in 16C leaf patches expressing HC-Pro mutants HC_FINK_, HC_FKNK_, and HC_FRNA_ were reduced significantly (*P* < 0.05) compared to those expressing wild type HC-Pro (**Figure 1F**). Interestingly, the HC-Pro accumulation levels in 16C leaf patches of mutants HC_FINK_, HC_FKNK_, and HC_FRNA_ were also reduced to a nearly undetectable level (**Figure 1F**).

These results showed that the residues R^184^ and K^186^ of FRNK motif played a critical role in the accumulation level and RSS activity of HC-Pro and virulence of SCMV in plants.

### Attenuated Mutant SCMV-HC_FINK_ Could Protect Maize Plants From Severe Infection

**Figure 2:**
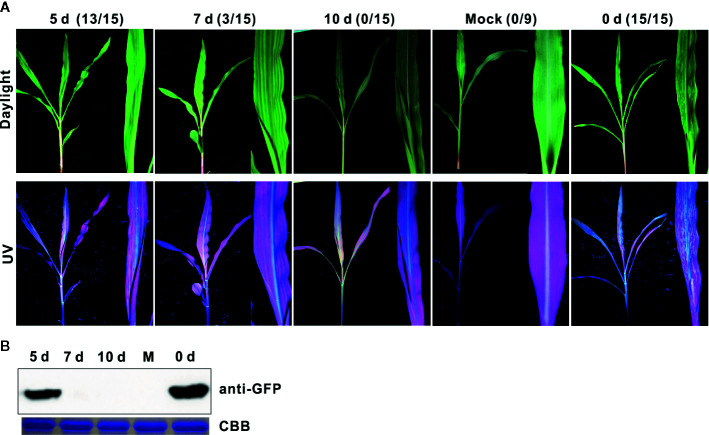
Cross protection effect of attenuated mutant SCMV-HC_FINK_ against severe infection. **(A)** Symptoms in maize plants challenged with SCMV-GFP at ten days post challenge inoculation with intervals of 0, 5, 7, or 10 dpi. Mock plants were inoculated with empty vector pCB301-Rz. The number of symptomatic/inoculated maize plants was listed in brackets. **(B)** The GFP accumulation levels of SCMV-GFP at ten days post challenge inoculation. CBB, Coomassie brilliant blue. The experiments were repeated three times independently.

To evaluate the cross-protection effect of SCMV-HC_FINK_ mutant, we first inoculate maize plants with an SCMV-HC_FINK_ mutant without the *gfp* reporter gene. For challenge inoculation, wild type SCMV with the *gfp* reporter gene (SCMV-GFP) was mechanically inoculated to the upper non-inoculated leaves at five, seven, and ten days interval. At ten days post challenge inoculation, mosaic symptom under daylight and strong GFP fluorescence under UV light were observed in all of the non-protected control maize plants, and 13 out of 15 protected maize plants at an interval of five days (**Figure 2A**); with an interval of seven days, only three out of 15 protected maize plants showed apparent mosaic and GFP fluorescence (**Figure 2A**). With an interval of ten days, no maize plant showed mosaic symptoms and GFP fluorescence after ten days post challenge inoculation (**Figure 2A**). Western blotting analysis showed the presence of GFP in the control and the treatments at five days post challenge inoculation, but not at seven and ten days when there were no symptoms observed in the maize plants (**Figure 2B**). These results indicated that attenuated mutant SCMV-HC_FINK_ could provide complete protection to the infection of wild type SCMV with an interval of ten days.

The HC-Pro coding sequences of the SCMV progeny at ten days post challenge inoculation were determined. The sequencing results showed that, with the interval of five days, the codons of the amino acid at position 184 were ATT (codon for I^184^) and CGA (codon for R^184^), indicating that mix infection of the mutant SCMV-FINK and wild type SCMV occurred in those maize plants; with the interval of ten days, the codon of the amino acid at position 184 was ATT (codon for I^184^) (**Supplemental Figure 2**), indicating the wild type virus was completely excluded by the FINK mutant.

### The Spontaneous Mutation of G^440^ to R Restored the Virulence of SCMV and RSS Activity of HC-Pro

**Figure 3:**
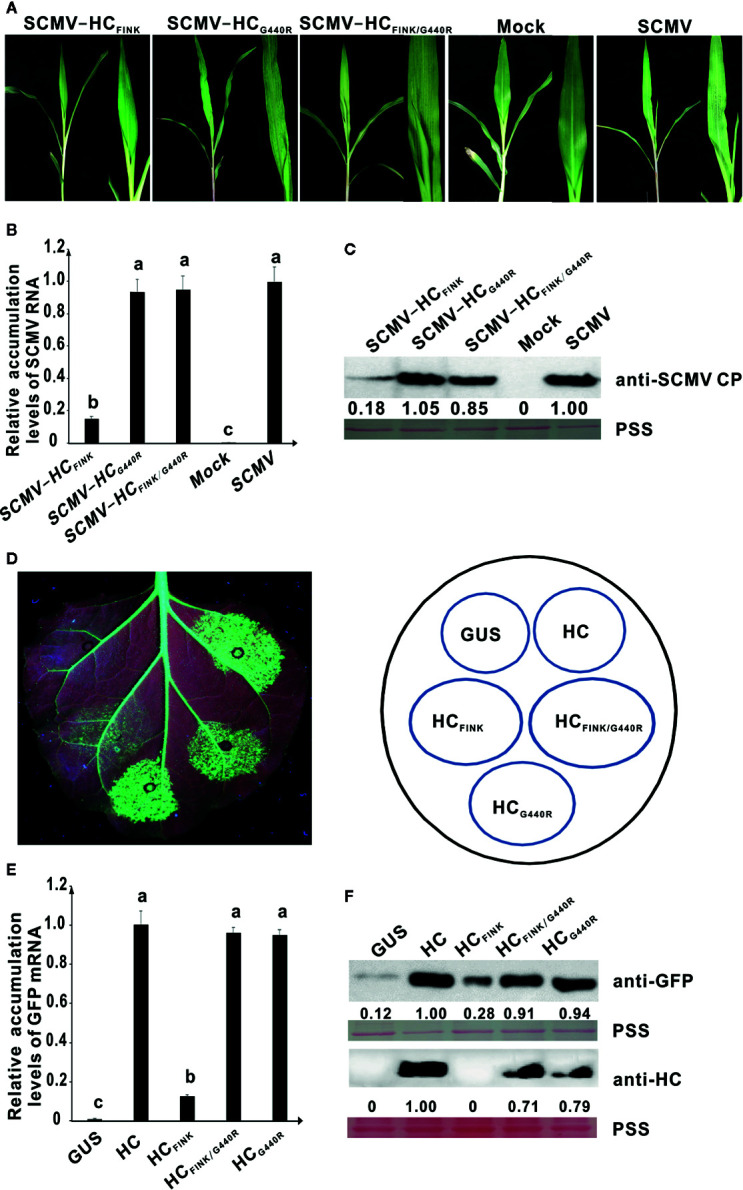
The spontaneous mutation of G^440^ to R restored the RSS activity of mutant HC-Pro and the virulence of attenuated SCMV. **(A)** Symptoms caused by wild type and mutant SCMV in maize plants at ten dpi. SCMV-HC_FINK_: SCMV mutant with substitution of R^184^ with I in FRNK motif of HC-Pro. SCMV-HC_G440R_: SCMV mutant with substitution of G^440^ with R in HC-Pro. SCMV-HC_FINK/G440R_: SCMV mutant with double substitutions of R^184^ with I and G^440^ with R. Mock, empty vector pCB301-Rz. SCMV, wild type SCMV. **(B)** The relative accumulation levels of wild type and mutant SCMV RNA in upper non-inoculated maize leaves at ten dpi. Different letters indicate significant differences (*P* < 0.05). **(C)** The accumulation levels of wild type and mutant SCMV in upper non-inoculated maize leaves at ten dpi. **(D)** RSS activity of wild type and mutant HC-Pro in *Agrobacterium* co-infiltration assay. HC_FINK_: HC-Pro mutant with substitution of R^184^ with I. HC_G440R_: HC-Pro mutant with substitution of G^440^ with R. HC_FINK/G440R_: HC-Pro mutant with substitutions of R^184^ with I and G^440^ with R. GUS, pBin-GUS as a negative control. HC, wild type HC-Pro. **(E)** The GFP mRNA accumulation levels in agroinfiltrated 16C leaf patches. Different letters indicate significant differences (*P* < 0.05). **(F)** Western blotting analysis of GFP and HC-Pro accumulation levels in agroinfiltrated leaf patches of 16C. Lower panel, Ponceau S staining (PSS) as a loading control. The experiments were repeated three times independently.

At 35 dpi, one of the ten maize plants infected with attenuated mutant SCMV-HC_FINK_ showed severe mosaic symptoms identical to those infected with wild type SCMV. We suspected the occurrence of a complementary mutation that restored the functionality of SCMV HC-Pro. The HC-Pro coding sequence from the SCMV progeny in this maize plant was sequenced and aligned. The results showed that the coding sequence for HC-Pro remained unchanged except that GGA, the codon for G at position 440 (G^440^) of HC-Pro_FINK_, mutated to AGA, which is the codon for R. To elucidate whether the amino acid mutation was responsible for the symptom restoration, we obtained mutated plasmids pSCMV-HC_FINK/G440R_ and pSCMV-HC_G440R_ using primers listed in **Supplemental Table 1**. At ten dpi, both SCMV-HC_FINK/G440R_ and SCMV-HC_G440R_ caused mosaic as severe as that of wild type SCMV, in the upper non-inoculated leaves of maize plants, while, as described above, SCMV-HC_FINK_ only induced mild mosaic symptoms (**Figure 3A**). These results indicated that mutation of G^440^ to R restored the virulence of attenuated mutant SCMV-HC_FRNR_. Results of qRT-PCR showed that the RNA accumulation levels of SCMV, SCMV-HC_FINK/G440R_, and SCMV-HC_G440R_ had no significant difference but were significantly higher (P < 0.05) than that of SCMV-HC_FINK_ (**Figure 3B**). Results of western blotting analysis showed that the accumulation levels of SCMV, SCMV-HC_FINK/G440R_, and SCMV-HC_G440R_ were at the same level, while that of SCMV-HC_FINK_ decreased by about 80% (**Figure 3C**).

To investigate the effects of G^440^ to R mutation on the RSS activity, site-directed mutations were introduced to pBin-HC and pBin-HC_FINK_ and produced plasmids pBin-HC_G440R_ and pBin-HC_FINK/G440R_. At three dpai, weak green fluorescence was observed in the 16C leaf patches expressing HC_FINK_ under UV light. In contrast, the green fluorescence in the 16C leaf patches expressing HC_FINK/G440R_ was as strong as that expressing wild type HC-Pro (**Figure 3D**), indicating that the G^440^ to R mutation restored the RSS activity of mutant HC_FINK_. The green fluorescence in the 16C leaf patches expressing HC_G440R_ was of the same level as that expressing wild type HC-Pro (**Figure 3D**). The results of qRT-PCR showed that the accumulation levels of GFP mRNA in 16C leaf patches expressing wild type HC-Pro, HC_G440R_, and HC_FINK/G440R_ were similar, all of which were significantly higher than that of HC_FINK_ (**Figure 3E**). Western blotting analysis showed that the GFP accumulation levels in 16C leaf patches expressing wild type HC-Pro, HC_G440R_, and HC_FINK/G440R_ had no significant difference; the accumulation levels of HC_G440R_ and HC_FINK/G440R_ amounted to 71 and 79%, respectively, of wild type HC-Pro, indicating that the mutation of G440 to R could rescue the accumulation levels of HC_FINK_ in 16C leaf patches (**Figure 3F**).

These results indicated that the spontaneous mutation of GGA (codon for G^440^) to AGA (codon for R) was a complementary mutation that could rescue the function of SCMV HC-Pro in virulence and RSS activity.

### The Mutation of G^440^ to R in HC-Pro Restored the Synergism of MCMV and SCMV

**Figure 4:**
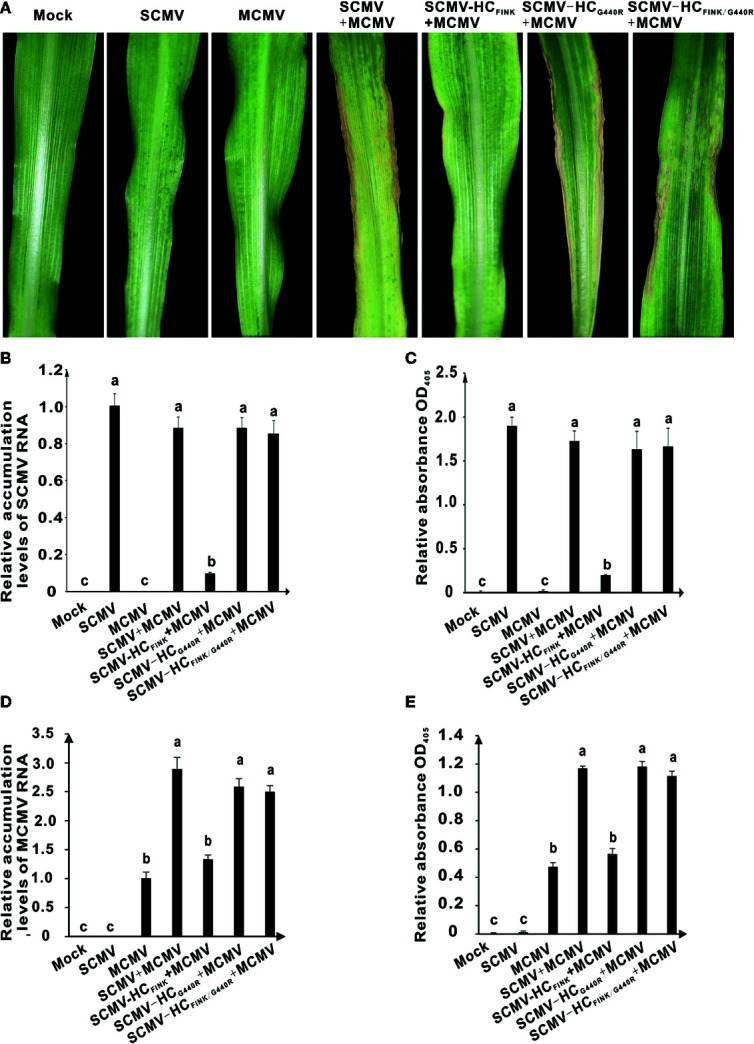
The spontaneous mutation of G^440^ to R restored the synergistic infection of MCMV and SCMV HC-Pro FINK mutant. **(A)** The symptoms on the first upper non-inoculated leaves of maize plants infected with MCMV, SCMV, MCMV and wild type or mutant SCMV at 12 dpi. **(B, D)** The relative accumulation levels of SCMV and MCMV RNA in maize leaves at nine dpi. **(C, E)** The accumulation levels of SCMV and MCMV in upper non-inoculated maize leaves at nine dpi. The experiments were repeated three times independently. The statistical analyses as above. Different letters indicate significant differences (*P* < 0.05).

SCMV has synergism with MCMV. The co-infection of SCMV and MCMV causes lethal necrosis of maize leaves (Mahuku et al., 2015; Xia et al., 2016). Next, we investigated the effects of substitution of R with I in FRNK motif of HC-Pro on the synergism of MCMV and SCMV and whether the mutation of G^440^ to R in HC-Pro could rescue the synergism. At 12 dpi, MCMV caused chlorotic mottling in maize plant; co-infection of SCMV and MCMV resulted in lethal necrosis on maize leaves and a dramatic increase in the accumulation levels of MCMV (**Figure 4A**). The co-infection of SCMV-HC_FINK_ and MCMV only induced severe mosaic symptom but no necrosis, indicating that mutation of R to I in FRNK motif of HC-Pro impaired the synergism between MCMV and SCMV. Maize plants infected with SCMV-HC_FINK/G440R_ and MCMV showed lethal necrosis symptoms (**Figure 4A**), indicating that the mutation of G^440^ to R in HC-Pro rescued the synergism between MCMV and SCMV. Results of qRT-PCR and ELISA showed that co-infection of MCMV and SCMV had no significant effect on the accumulation levels of SCMV (**Figures 4B, C**) but increased those of MCMV significantly (**Figures 4D, E**). The accumulation levels of MCMV in plants co-infected with MCMV and SCMV-HC_FINK_ were at the same level with those in plants infected with MCMV only; however, those in plants co-infected with MCMV and SCMV-HC_FINK/G440R_ increased to the same level of co-infection with MCMV and wild type SCMV (**Figures 4D, E**). These results indicated that the complementary mutation of G^440^ to R in HC-Pro could rescue the synergism of MCMV and SCMV.

### Mutation of G^440^ to R or K, but Not H, Could Restore the Function of SCMV HC-Pro in Virulence and RNA Silencing Suppression Activity

**Figure 5:**
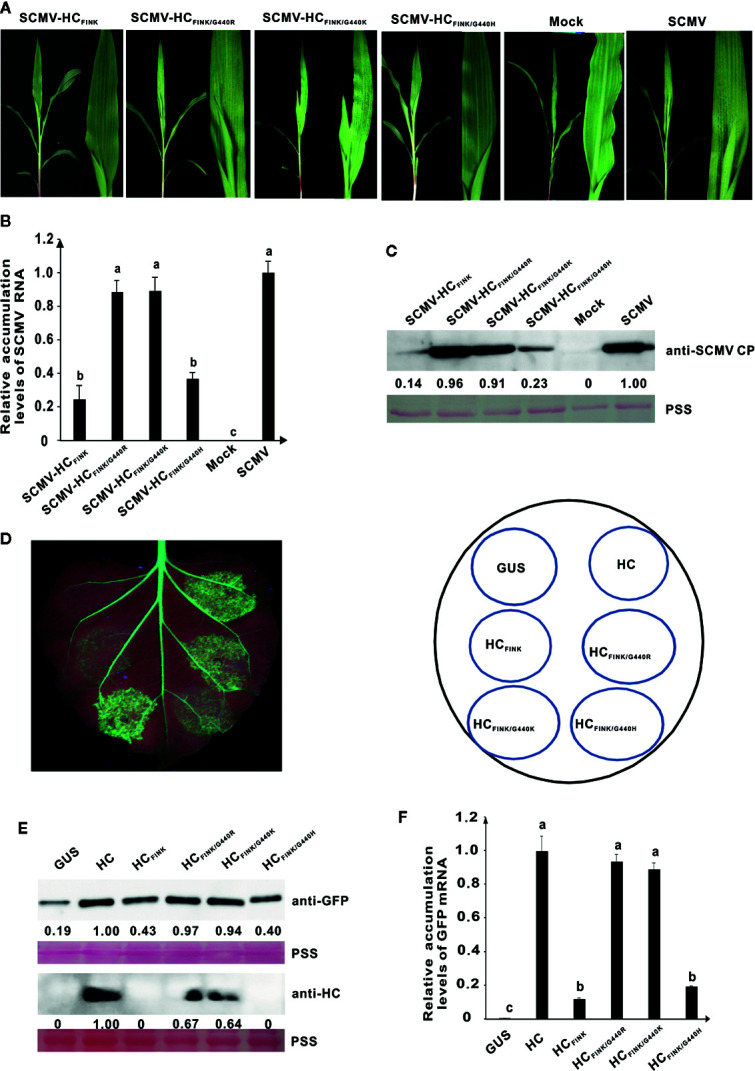
The mutation of G^440^ to K also restored the RSS activity of mutant HC-Pro and the virulence of attenuated SCMV. **(A)** Symptoms caused by wild type and mutant SCMV in maize plants at ten dpi. SCMV-HC_FINK_: SCMV mutant with mutation of R^184^ to I in FRNK motif of HC-Pro. SCMV-HC_FINK/G440R_, SCMV-HC_FINK/G440K_, and SCMV-HC_FINK/G440H_: SCMV mutants with additional mutation of G^440^ to R, K, and H, respectively, besides R^184^ to I, in HC-Pro. Mock, empty vector pCB301-Rz. SCMV, wild type SCMV. **(B)** The relative accumulation levels of wild type and mutant SCMV RNA in upper non-inoculated maize leaves at ten dpi. Different letters indicate significant differences (*P* < 0.05). **(C)** The accumulation levels of wild type and mutant SCMV in upper non-inoculated maize leaves at ten dpi. **(D)** RSS activity of wild type and mutant HC-Pro in *Agrobacterium* co-infiltration assay. The G^440^ in HC_FINK_ was substituted with R, K, and H in HC_FINK/G440R_, HC_FINK/G440K_, and HC_FINK/G440H_, respectively. GUS, negative control. HC, wild type HC-Pro. **(E)** Western blotting analysis of GFP and HC-Pro accumulation levels in agroinfiltrated leaf patches of 16C. Lower panel, Ponceau S staining (PSS) as a loading control. **(F)** The GFP mRNA accumulation levels in agroinfiltrated 16C leaf patches. The experiments were repeated three times independently. The statistical analyses as above. Different letters indicate significant differences (P < 0.05).

To further elucidate whether the net-charge of HC-Pro is responsible for RSS activity and virulence, we constructed plasmids pSCMV-HC_FINK/G440K_ and pSCMV-HC_FINK/G440H_ from which the G^440^ of HC-Pro in progeny viruses would mutate to K and H, respectively. At ten dpi, SCMV-HC_FINK/G440K_ induced mosaic symptom identical with that of SCMV and SCMV-HC_FINK/G440R_ in the upper non-inoculated maize leaves; however, SCMV-HC_FINK/G440H_ induced only mild mosaic symptoms as those of SCMV-HC_FINK_ (**Figure 5A**). Results of qRT-PCR and western blotting showed that the accumulation levels of SCMV, SCMV-HC_FINK/G440R_, and SCMV-HC_G440K_ had no significant difference but were significantly higher (*P*<0.05) than those of SCMV-HC_FINK_ and SCMV-HC_FINK/G440H_ (**Figures 5B, C**). These results indicated that mutation of G^440^ to K, but not H, could restore the function of SCMV HC-Pro in virulence.

Mutations of G^440^ to R and H were introduced to SCMV HC-Pro expressing vectors. In RSS assay, the green fluorescence in 16C leaf patches expressing HC_FINK/G440K_ were as strong as HC_FINK/G440R_, while those in leaf patches expressing HC_FINK/G440H_ were marginally stronger than HC_FINK_ (**Figure 5D**), indicating that mutation of G^440^ to K, but not H, could restore the RSS function of SCMV HC-Pro FINK mutant. The accumulation levels of GFP in the infiltrated 16C leaf patches were consistent with the result of green fluorescence (**Figure 5E**). Western blotting analysis also showed that HC_FINK/G440K_ and HC_FINK/G440R_ could accumulate to 64 and 67%, respectively of wild type HC-Pro, while both HC_FINK/G440H_ and HC_FINK_ were under the detection threshold with SCMV HC-Pro antibody (**Figure 5E**). The qRT-PCR analysis showed that the GFP mRNA in 16C leaf patches expressing HC_FINK/G440K_ and HC_FINK/G440R_ could accumulate to levels similar to wild type HC-Pro, while the GFP mRNA accumulation level of HC_FINK/G440H_ was at the same level as HC_FINK_ (**Figure 5F**). These results indicated that mutation of G^440^ to R or K, but not H, could restore the RSS activity and accumulation levels of SCMV HC-Pro.

Taken together, these results indicated that the virulence and accumulation levels had no relationship with the net charge of SCMV HC-Pro.

### Mutations on Corresponding Amino Acid of G^440^ Restored Functions of WMV and TVBMV HC-Pro in Virulence and RNA Silencing Suppression

Sequence alignment showed that the amino acid corresponding to G^440^ of SCMV HC-Pro was not a conserved amino acid (Rodamilans et al., 2018). The amino acid corresponding to G^440^ of SCMV HC-Pro was N^437^ for WMV HC-Pro and S^438^ for TVBMV HC-Pro (**Supplemental Figure 3**). To test the role of these two amino acids on RSS activity and viral virulence, we introduced mutation to *gfp* gene-carrying infectious clones of WMV and TVBMV, producing mutant plasmids pWMV-HC_FINK_, pWMV-HC_N437R_, pWMV-HC_FINK/N437R_, pTVBMV-HC_FINK_, pTVBMV-HC_S438R_, and pTVBMV-HC_FINK/S438R_, respectively.

**Figure 6:**
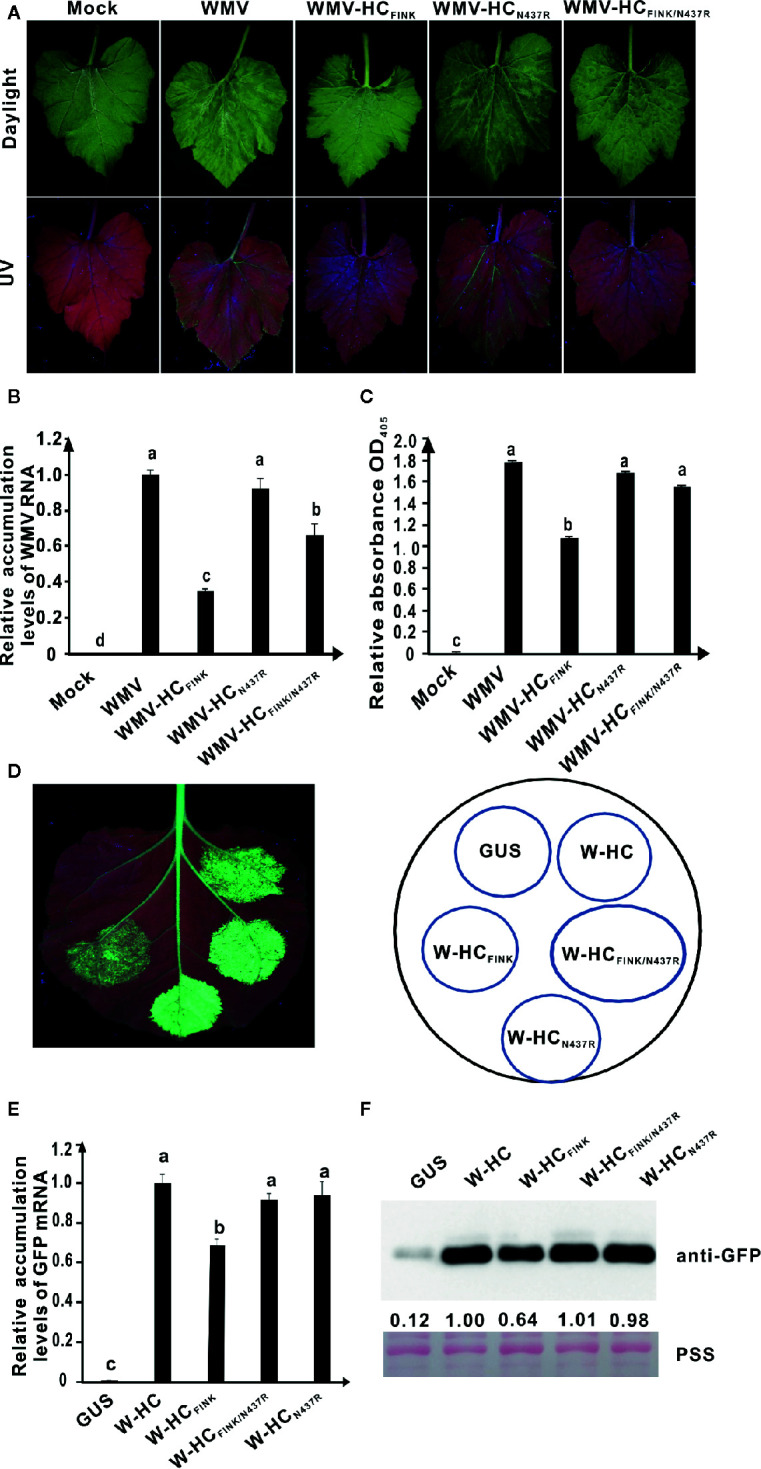
The substitution of N^437^ to R rescued the RSS activity of HC-Pro and virulence of watermelon mosaic virus (WMV). **(A)** Symptoms caused by wild type and mutant WMV in *Cucumis melo* plants at 15 dpi. The R was mutated to I in HC-Pro of WMV-HC_FINK_. The N^437^ in HC-Pro of WMV-HC_FINK_ was substituted with R in HC-Pro of WMV-HC_FINK/N437R_ and WMV-HC_N437R_. Mock, empty vector pCB301-Rz. WMV, wild type WMV. **(B)** The relative accumulation levels of wild type and mutant WMV RNA in upper non-inoculated *C. melo* leaves at 15 dpi. **(C)** The accumulation levels of wild type and mutant WMV in *C. melo* upper non-inoculated leaves at 15 dpi. **(D)** RSS activity of wild type and mutant HC-Pro in *Agrobacterium* co-infiltration assay. W-HC_FINK_: Mutant of WMV HC-Pro with mutation of R in FRNK motif to I. W-HC_N437R_: Mutant of WMV HC-Pro with mutation of N^437^ to R. W-HC_FINK/N437R_: Mutant of WMV HC-Pro with mutations of both R to I and N^437^ to R. GUS, negative control. W-HC, wild type WMV HC-Pro. **(E)** The GFP mRNA accumulation levels in agroinfiltrated 16C leaf patches as determined by qRT-PCR analysis. **(F)** Western blotting analysis of GFP accumulation levels in agroinfiltrated leaf patches of 16C using an antibody against GFP. Lower panel, Ponceau S staining (PSS) as a loading control. The experiments were repeated three times independently. The statistical analyses as above. Different letters indicate significant differences (*P* < 0.05).

The *Cucumis melo* plants were inoculated with pCB301-Rz empty vector, pWMV-HC_FINK_, pWMV-HC_N437R_, and pWMV-HC_FINK/N437R_, respectively. At 15 dpi, wild type WMV induced severe mosaic in the systematic leaves and green fluorescence under UV light; mutant WMV-HC_FINK_ induced drastically attenuated symptom and reduced green fluorescence under UV light; double mutant WMV-HC_FINK/N437R_ caused mosaic as severe as wild type WMV though the strength of green fluorescence was slightly weaker (**Figure 6A**). Results of qRT-PCR showed that the RNA accumulation level of WMV-HC_FINK/N437R_ was lower than those of WMV-HC_N437R_ and wild type WMV but significantly higher than that of WMV-HC_FINK_ (**Figure 6B**). Western blotting results showed that the accumulation levels of WMV-HC_FINK/N437R_, WMV-HC_N437R_, and wild type WMV were at a similar level, and significantly (*P* < 0.05) higher than that of WMV-HC_FINK_ (**Figure 6C**). These results indicated that mutation of N^437^ to R restored the virulence of attenuated mutant WMV-HC_FINK_.

To investigate the RSS activity of WMV HC-Pro mutants, we conducted site-directed mutagenesis on pBin-W-HC, a plasmid expressing WMV HC-Pro gene. The resultant plasmids pBin-W-HC_FINK_, pBin-W-HC_N437R_, and pBin-W-HC_FINK/N437R_, respectively, were used in agroinfiltration assay. At five dpai, weak green fluorescence was observed in 16C leaf patches expressing W-HC_FINK_ (W-, short for WMV) under UV light; the green fluorescence in the 16C leaf patches expressing W-HC_FINK/N437R_ was as strong as that expressing wild type WMV HC-Pro (W-HC) (**Figure 6D**), indicating that mutation of N^437^ to R restored the RSS activity of mutant HC-Pro. The green fluorescence in the 16C leaf patches expressing HC_N437R_ was same as that in the 16C leaf patches expressing W-HC (**Figure 6D**). Results of qRT-PCR showed that the accumulation levels of GFP mRNA in 16C leaf patches expressing W-HC, W-HC_N437R_, and W-HC_FINK/N437R_ were similar and significantly higher (*P* < 0.5) than that of W-HC_FINK_ (**Figure 6E**). The western blotting analysis showed that the GFP accumulation levels in 16C leaf patches expressing W-HC, W-HC_N437R_, and W-HC_FINK/N437R_ had no significant difference (**Figure 6F**).

**Figure 7:**
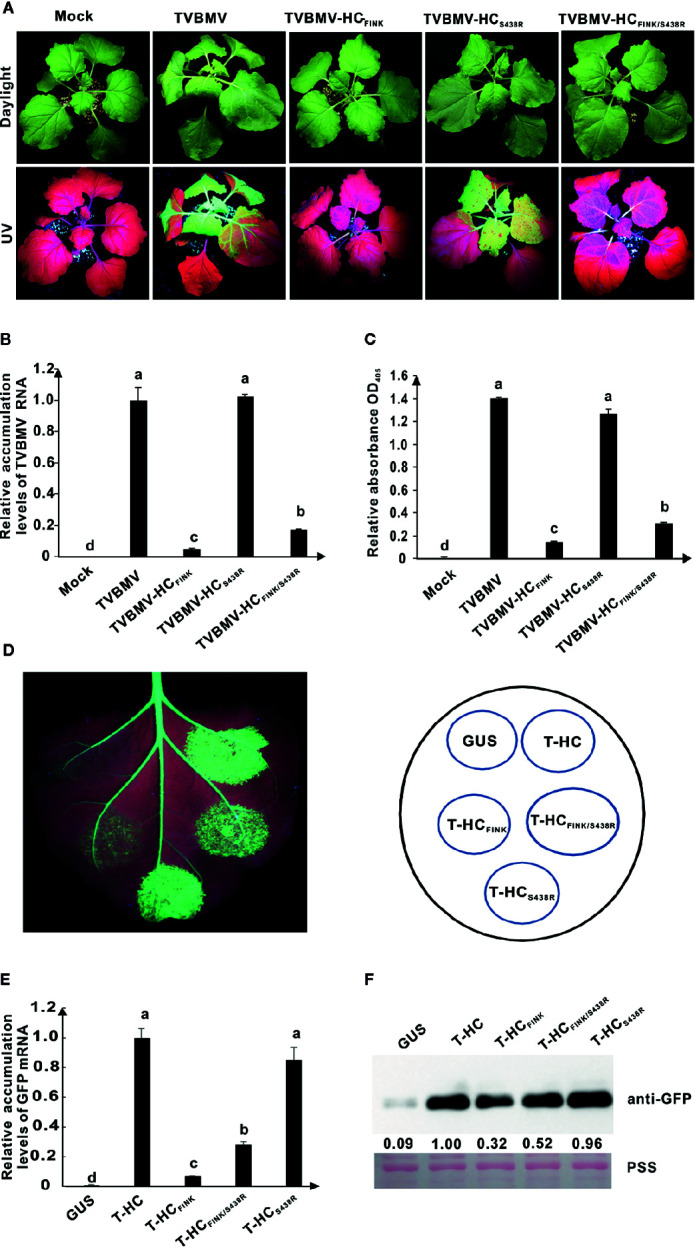
The substitution of S^438^ to R rescued the HC-Pro RSS activity and virulence of TVBMV. **(A)** Symptoms caused by wild type and mutant TVBMV in *N. benthamiana* plants at ten dpi. The R in FRNK motif was mutated to I in HC-Pro of TVBMV-HC_FINK_. The S^438^ in HC-Pro was substituted with R in HC-Pros of TVBMV-HC_FINK/S438R_ and TVBMV-HC_S438R_. Mock, empty vector pCB301-Rz. TVBMV, wild type TVBMV. **(B)** The relative accumulation levels of wild type and mutant TVBMV RNA in upper non-inoculated *N. benthamiana* leaves at ten dpi. **(C)** The accumulation levels of wild type and mutant TVBMV in *N. benthamiana* upper non-inoculated leaves at ten dpi. **(D)** RSS activity of wild type and mutant HC-Pro in *Agrobacterium* co-infiltration assay. The R in FRNK motif was mutated to I in HC_FINK_. The S^438^ was substituted with R in HC_FINK/S438R_ and HC_S438R_. GUS, negative control. HC, wild type HC-Pro. **(E)** The GFP mRNA accumulation levels in agroinfiltrated 16C leaf patches. **(F)** Western blotting analysis of GFP accumulation levels in agroinfiltrated leaf patches of 16C. Lower panel, Ponceau S staining (PSS) as a loading control. The experiments were repeated three times independently. The statistical analyses as above. Different letters indicate significant differences (*P* < 0.05).

At ten dpi, TVBMV and TVBMV-HC_S438R_ induced typical mosaic and epinasty in the upper non-inoculated leaves and stunting of the *N. benthamiana* plants, TVBMV-HC_FINK_ caused very slight mosaic symptom, and TVBMV-HC_FINK/S438R_ caused mild vein clearing and mosaic symptoms (**Figure 7A**). Under UV light, green fluorescence in the upper non-inoculated leaves of *N. benthamiana* plants was positively correlated with the severity of symptoms (**Figure 7A**). The results of qRT-PCR and ELISA showed that the accumulation levels of TVBMV-HC_FINK/S438R_ were higher than those of TVBMV-HC_FINK_ but significantly lower than those of TVBMV and TVBMV-HC_S438R_ (**Figures 7B, C**). In RSS assay, the 16C leaf patches expressing T-HC_FINK/S438R_ (T-, short for TVBMV) showed stronger green fluorescence than the patches expressing T-HC_FINK_ at three dpai (**Figure 7D**). The results of qRT-PCR and western blotting analyses confirmed this conclusion (**Figures 7E, F**). These results indicated that additional mutation of S^438^ to R could restore TVBMV virulence and enhance the RSS function of TVBMV HC-Pro FINK mutant.

## Discussion

In this study, we showed that the mutation of R^184^ in the conserved FRNK motif of HC-Pro affected its accumulation level and RSS activity, the virulence and synergism with MCMV of SCMV, all of which could be rescued by a complementary mutation of G^440^ to R; similar substitution of WMV and TVBMV HC-Pro could also rescue their RSS activity and restore the virulence of both viruses.

RNA silencing is a highly conserved sequence-specific regulatory mechanism in eukaryotic cells and plays a key role in antiviral activities in many organisms (Bronkhorst and van Rij, 2014; Huang et al., 2014; Zhang C. et al., 2015). To counter the host defense mechanism, many viruses encode RNA silencing suppressors during the long-term co-evolution with hosts. HC-Pro is the RNA silencing suppressor and the virulence determinant of potyviruses and is also involved in the final yield of viral particles (Anandalakshmi et al., 1998; Kasschau and Carrington, 1998; Valli et al., 2014). Many studies have shown that the RSS activity of HC-Pro is correlated with virus accumulation and symptom expression. Substitution of I for R in the conserved FRNK motif of HC-Pro affected the symptom caused by ZYMV, TVBMV, and TuMV (Gal-On, 2000; Shiboleth et al., 2007; Gao et al., 2012). Mutations of threonine (T) at position 27 to I and D at position 193 to Y located in a putative zinc finger domain of HC-Pro reduced its RSS activity and virulence of clover yellow vein virus (Yambao et al., 2008). Mutations in the conserved CDNQLD motif located 12 amino acids downstream from the FRNK motif in HC-Pro reduced the virulence and accumulations of ZYMV (Desbiez et al., 2010). In this study, we showed that mutations on R^184^ and K^186^ of FRNK motif in HC-Pro affected the RSS activity and virulence of SCMV (**Figure 1**); substitution of I for R in the FRNK motif of WMV HC-Pro reduced its RSS activity and the symptom severity caused by mutant WMV (**Figures 6A, D**). The decrease in the virulence of SCMV and WMV mutants may be due to the change in the HC-Pro RSS activity. The silencing deficient HC-FINK mutant of TEV could not suppress GFP silencing or bind detectable virus-derived small RNAs duplex, but lead to silencing of the HC-Pro construct itself and reduced expression of HC-Pro (Shiboleth et al., 2007). The mutation of R184 to I, K or K186 to A significantly decreased the accumulation levels of GFP and HC-Pro in the 16C leaf patches (**Figures 1D–F**), indicating the mutants of SCMV HC-Pro were unstable in the plants. The additional mutation of G440 to R could rescue the accumulation levels of HC_FINK_ in 16C leaf patches (**Figure 3F**). We also investigated the accumulation levels of SCMV HC-Pro and its variants HC_FINK_, HC_G440R_, and HC_FINK/G440R_ in *E. coli* cells. The accumulation levels of wild type HC-Pro, mutants HC_FINK_, HC_G440R_, and HC_FINK/G440R_ showed no significant difference, indicating HC-Pro and its mutants are stable in *E. coli* cells (**Supplemental Figure 4**). These results showed that the stability and accumulation of HC-Pro and its mutants in plants were different from those in *E. coli*, probably due to the degradation of autophagy in plants (Nakahara et al., 2012).

The nature, including the net charge, of amino acids in HC-Pro is speculated to be critical for ZYMV virulence (Kimalov et al., 2004; Chiang et al., 2007). In this paper, the substitution of two positively-charged amino acids with neutral one in FRNK motif reduced the RSS activity of HC-Pro and the virulence of SCMV (**Figure 1**); furthermore, the spontaneous complementary mutation was from the neutral amino acid G to positively-charged R (**Figure 3**). All these results seem to indicate that the net charge of HC-Pro plays a critical role in the HC-Pro RSS activity and virulence of SCMV. However, the substitution of G with positively-charged H did not have a similar phenotype (**Figure 5**). Therefore, we concluded that the primary sequence and probably their higher structure, instead of net charge, account for the function of HC-Pro in these processes. These results provide novel evidence for the complementary mutation of potyviruses in maintaining the HC-Pro RSS activity and potyviral virulence.

Synergistic interactions between viruses from the genus *Potyvirus* and other genera have been well documented. In most cases, the beneficiary of synergistic interaction is the virus from the genus other than *Potyvirus* (Karyeija et al., 2000; Pacheco et al., 2012; Zhou et al., 2017). However, in the case of sweet potato feathery mottle virus (SPFMV, genus *Potyvirus*) and sweet potato chlorotic stunt virus (SPCSV, genus *Crinivirus*), SPFMV is the beneficiary (Karyeija et al., 2000; Untiveros et al., 2007). The synergistic infection of maize plants with MCMV and SCMV induces severe mosaic and lethal necrosis and results in a dramatic increase in the accumulation levels of MCMV (Xia et al., 2016). Our results further showed that substitutions of R with I in FRNK motif of HC-Pro abolished the synergism of SCMV and MCMV, and the complementary mutation of G^440^ to R in FINK mutant restored the RSS activity of HC-Pro and the synergism of SCMV and MCMV (**Figure 4**).

The polymerases of RNA viruses lack the mechanism of proofreading and repair. This error-prone nature results in rapid genetic changes and the evolution of RNA viruses (Roossinck, 1997). Mutations occur continuously and randomly. Many mutations obtained are deleterious or lethal for viruses (García-Arenal et al., 2003). Some mutations will increase the fitness and competitiveness of progeny viruses. Potyviral HC-Pro plays an important role in compensatory molecular evolution which restores the wild type level of suppressor activity (Torres-Barceló et al., 2008; Torres-Barceló et al., 2009; Ambrós et al., 2018). The SCMV variant carrying the HC-Pro silencing deficient R184I mutation (reduction of 70% in silencing suppression activity) introduces a compensatory mutation of G^440^ to R in HC-Pro, which restored the RSS activity of HC-Pro and increased the accumulation levels of SCMV (**Figure 3**). The compensatory mutation will endow the attenuated mutants more competitive advantage. Sequence alignment of fifteen potyviruses HC-Pro showed that G^440^ in SCMV HC-Pro was not a conservative amino acid (**Figures S3** and **S5**). The corresponding amino acid at position 440 was highly variable in that the amino acid corresponding to G^440^ of SCMV HC-Pro was S^438^ for sweet potato feathery mottle virus, and E^438^ for TuMV (Rodamilans et al., 2018). The non-conservative amino acids enable more options for potyviruses and increase their fitness and competitiveness (Haikonen et al., 2013). Theoretically, mutants SCMV-HC_FKNK_ and SCMV-HC_FRNA_ would introduce compensatory mutations to improve viral fitness. But within the experiment period of 35 days, the maize plants inoculated with attenuated mutants SCMV-HC_FKNK_ and SCMV-HC_FRNA_ did not generate compensatory mutations. The amino acids at position 192 of potyviral HC-Pro were not conserved. Mutation of valine (V^192^) to A reduced the RNA silencing suppression activity of TEV HC-Pro by 30%. A compensatory mutation in position 442 restored the RSS activity, but not the virulence of TEV (Torres-Barceló et al., 2009). All these results show a need for extensive evaluation of attenuated mutants before applying them in cross-protection of plants.

We built the 3D structure of the cysteine protease domain (CPD) of SCMV HC-Pro (from aa 338 to 460) by homology modeling (Guo et al., 2011). It showed that G^440^ of SCMV HC-Pro (HC-WT) was located inside an *α*-helix, while the side chain of R from mutant HC-G440R formed two hydrogen bonds with the side chain of N^403^ in the adjacent *α*-helix (2.0 and 3.1 Å, respectively); the side chain of K from mutant HC-G440K formed one hydrogen bond with N^403^, but the side chain of H from mutant HC-G440H was too far away from N^403^ (6.8 and 7.2 Å) to form any hydrogen bond (**Supplemental Figure 6**). Whether the mutant could form hydrogen bond was consistent with their function in the reversal of virulence and RNA silencing suppression activity. But the 3D structure of the complete HC-Pro is not available. The 3D structures mentioned above did not include the critical amino acid R^184^ in FRNK. The predicted 3D structures of SCMV HC-Pro CPD could not explain the full story why mutation of G^440^ to R could complement the function of SCMV HC-Pro.

The mechanisms of cross-protection are not fully understood. RNA silencing was used to be a common explanation for cross-protection. However, cross-protection works in the plant mutants with two or three *dcl* genes (encoding DICER-like proteins which are essential components for RNA silencing) knocked-out (Ziebell and Carr, 2009; Zhang X. F. et al., 2015), implying cross-protection manifests an mechanism that is distinct from RNA silencing. Co-infected viruses (with no synergism) or virus isolates remained predominantly separate in the infected plants (Dietrich and Maiss, 2003; Takeshita et al., 2004). In our cross-protection assay, maize plants were ‘protected’ with attenuated mutant without the *gfp* gene, and challenged with SCMV with *gfp* gene (SCMV-GFP). Therefore, the existence of wild type virus can be detected in the upper non-inoculated leaves by green fluorescence under UV light and western blotting with antibody against GFP. With an interval of five days, both wild type SCMV and SCMV-FINK could be detected in upper non-inoculated leaves (**Figures 2B**, **S2**, and **S7**), indicating that SCMV-FINK could not provide protection against wild type SCMV. However, with an interval of ten days, no wild type SCMV RNA or GFP could be detected in the upper non-inoculated leaves of maize plants (**Figures 2B**, **S2**, and **S7**), indicating that the mutant SCMV-HC_FINK_ could provide complete protection against wild type SCMV-GFP with an interval of ten days. These results give further evidence that cross-protection is linked to superinfection resistance (Zhang X. F. et al., 2015).

The safety of attenuated mutants is the primary consideration for the potential field application; however, studies are limited. The GAC mutant of ZYMV was stable after nine passages through squash plants after nine months (Lin et al., 2007). The TuMV HC-Pro mutant with substitution of R to K is as stable as wild type one *in planta*, and the attenuated virus mutant Tu-GK induced stable symptoms in *N. benthamiana* and was symptomless in *Arabidopsis* for more than four passages at an interval of seven days (Kung et al., 2014). The symptom caused by and genome of two PVX mutants were stable through five passages covering a span of 50 days (Cong et al., 2019). Here, the attenuated mutant SCMV-HC_FINK_ could protect maize plants from a severe infection of wild type SCMV (**Figure 2**); however, a complementary mutation of G^440^ to R occurred within 35 days post inoculation and restored the virulence of SCMV-HC_FINK_ mutant, RSS activity of HC-Pro, and synergism of SCMV and MCMV. Then we did not conduct cross-protection tests with other two mutants, though no complementary mutation occurred within 35 days (data not shown). More efforts are necessary to assess and ensure the stability of such attenuated mutants before considering the possibility of their application in practice.

To sum up, our results reveal the linkage between the HC-Pro function in RSS activity, virulence, synergism, and the reversal of HC-Pro roles by a spontaneous complementary mutation. These results increase our understanding of the evolution of potyviruses and provide a theoretical guide for the prevention and control of SCMV by cross-protection. The study also shows the need for complete and proper evaluation of attenuated mutants before application of cross-protection in the fields.

## Data Availability Statement

All datasets presented in this study are included in the article/**Supplementary Material**.

## Author Contributions

X-JX., H-GL, and X-DL conceived the project and designed this work. X-JX and CG performed the experiments. X-JX., D-JC, CG, and Y-PT conducted bioinformatics analyses. X-JX, CG, and LZ-L provided experimental materials. All authors analyzed and reviewed experimental data. X-JX., Y-PT and X-DL wrote the paper. All authors contributed to the article and approved the submitted version.

## Funding

This work was supported by funds from the National Key Research and Development Program (2018YFD0200604), Shandong Modern Agricultural Technology & Industry System (SDAIT-02-10), Shandong ‘Double Top’ Program (SYL2017XTTD11), and ‘Taishan Scholar’ Construction Project (TS201712023).

## Conflict of Interest

The authors declare that the research was conducted in the absence of any commercial or financial relationships that could be construed as a potential conflict of interest.

## Supplementary Material

Figure S1 Alignment of the FRNK motif in HC-Pros of six potyviruses. The red triangles pointed to the amino acids (numbered as in SCMV HC-Pro) for mutation.

Figure S2 The HC-Pro coding sequences of the SCMV progeny. The maize plants were challenge inoculated with intervals of 5, 7, and 10 days. The upper non-inoculated (systemic) maize leaves were collected at ten days post challenge inoculation. The codons of the amino acid at position 184 in SCMVHC-Pro were underlined in red.

Figure S3 Alignment of partial HC-Pro amino acid sequences of SCMV, WMV, and TVBMV. The corresponding amino acids for G440 in SCMV HC-Pro (N437 in WMV HC-Pro and S438 in TVBMVHC-Pro) were indicated in the red box.

Figure S4 The stability of SCMV HC-Pro and its mutants in *E. coli.* Purified maltose binding protein (MBP)-tagged HC-Pro proteins were separated and stained by Coomassie brilliant blue. M, marker.

Figure S5 Alignment of HC-Pro amino acid sequences from fifteen potyviruses. The corresponding amino acids for G440 in SCMV HC-Pro were indicated in the red box.

Figure S6 The 3D structures of the cysteine protease domain of SCMVHC-Pro and its mutants(from aa 338-460). HC-G440R, HC-G440K and HC-G440H: SCMV HC-Pro mutants with additional mutation of G440 to R, K, and H, respectively. HC-WT, wild type HC-Pro.

Figure S7 The GFP accumulation levels of SCMV-GFP at ten days post challenge inoculation.CBB, Coomassie brilliant blue. The experiments were repeated three times independently.

## References

[B1] Ambrós S. de la Iglesia F. Rosario S. M. Butković A. Elena S. F. Abergel C. (2018). Engineered functional redundancy relaxes selective constraints upon endogenous genes in viral RNA genomes. Genome Biol. Evol. 10, 1823–1836. 10.1093/gbe/evy141 29982435 PMC6059116

[B2] Anandalakshmi R. Pruss G. J. Ge X. Marathe R. Mallory A. C. Smith T. H. (1998). A viral suppressor of gene silencing in plants. Proc. Natl. Acad. Sci. U. S. A. 95, 13079–13084. 10.1073/pnas.95.22.13079 9789044 PMC23715

[B3] Bance V. B. (1991). Replication of potato virus X RNA is altered in coinfections with potato virus Y. Virology 182, 486–494. 10.1016/0042-6822(91)90589-4 2024486

[B4] Bronkhorst A. W. van Rij R. P. (2014). The long and short of antiviral defense: small RNA-based immunity in insects. Curr. Opin. Virol. 7, 19–28. 10.1016/j.coviro.2014.03.010 24732439

[B5] Chen H. Cao Y. Li Y. Xia Z. Xie J. Carr J. P. (2017). Identification of differentially regulated maize proteins conditioning *Sugarcane mosaic virus* systemic infection. New Phytol. 215, 1156–1172. 10.1111/nph.14645 28627019

[B6] Cheng D. J. Tian Y. P. Geng C. Guo Y. Jia M. A. Li X. D. (2020). Development and application of a full-length infectious clone of potato virus Y isolate belonging to SYR-I strain. Virus Res. 276, 197827. 10.1016/j.virusres.2019.197827 31785306

[B7] Chiang C. H. Lee C. Y. Wang C. H. Jan F. J. Lin S. S. Chen T. C. (2007). Genetic analysis of an attenuated *Papaya ringspot virus* strain applied for cross-protection. Eur. J. Plant Pathol. 118, 333–348. 10.1007/s10658-007-9130-z

[B8] Chung B. Y. W. Miller W. A. Atkins J. F. Firth A. E. (2008). An overlapping essential gene in the *Potyviridae* . Proc. Natl. Acad. Sci. U. S. A. 105, 5897–5902. 10.1073/pnas.0800468105 18408156 PMC2311343

[B9] Cong Q. Wang Y. Liu J. Lan Y. Guo Z. Yang J. (2019). Evaluation of *Potato virus X* mild mutants for cross protection against severe infection in China. Virol. J. 16, 36. 10.1186/s12985-019-1143-7 30894176 PMC6425663

[B10] Cook G. van Vuuren S. P. Breytenbach J. H. J. Burger J. T. Maree H. J. (2016). Expanded strain-specific RT-PCR assay for differential detection of currently known citrus tristeza virus strains: a useful screening tool. J. Phytopathol. 164, 847–851. 10.1111/jph.12454

[B11] Desbiez C. Girard M. Lecoq H. (2010). A novel natural mutation in HC-Pro responsible for mild symptomatology of *Zucchini yellow mosaic virus* (ZYMV, Potyvirus) in cucurbits. Arch. Virol. 155, 397–401. 10.1007/s00705-009-0569-4 20112122

[B12] Dietrich C. Maiss E. (2003). Fluorescent labelling reveals spatial separation of potyvirus populations in mixed infected Nicotiana benthamiana plants. J. Gen. Virol. 84, 2871–2876. 10.1099/vir.0.19245-0 13679622

[B13] Folimonova S. Y. (2013). Developing an understanding of cross-protection by *Citrus tristeza virus* . Front. Microbiol. 4, 76. 10.3389/fmicb.2013.00076 23577008 PMC3616238

[B14] Gal-On A. (2000). A point mutation in the FRNK motif of the potyvirus helper component-protease gene alters symptom expression in cucurbits and elicits protection against the severe homologous virus. Phytopathology 90, 467–473. 10.1094/PHYTO.2000.90.5.467 18944551

[B15] Gao B. Cui X. W. Li X. D. Zhang C. Q. Miao H. Q. (2011). Complete genomic sequence analysis of a highly virulent isolate revealed a novel strain of *Sugarcane mosaic virus* . Virus Genes 43, 390–397. 10.1007/s11262-011-0644-2 21833715

[B16] Gao R. Tian Y. P. Wang J. Yin X. Li X. D. Valkonen J. P. (2012). Construction of an infectious cDNA clone and gene expression vector of *Tobacco vein banding mosaic virus* (genus *Potyvirus*). Virus Res. 169, 276–281. 10.1016/j.virusres.2012.07.010 22820405

[B17] García-Arenal F. Fraile A. Malpica J. M. (2003). Variation and evolution of plant virus populations. Int. Microbiol. 6, 225–232. 10.1007/s10123-003-0142-z 13680390

[B18] Geng C. Wang H. Y. Liu J. Yan Z. Y. Tian Y. P. Yuan X. F. (2017). Transcriptomic changes in *Nicotiana benthamiana* plants inoculated with the wild-type or an attenuated mutant of *Tobacco vein banding mosaic virus* . Mol. Plant Pathol. 18, 1175–1188. 10.1111/mpp.12471 27539720 PMC6638280

[B19] Gonzalez-Jara P. Atencio F. A. Martinez-Garcia B. Barajas D. Tenllado F. Diaz-Ruiz J. R. (2005). A single amino acid mutation in the plum pox virus helper component-proteinase gene abolishes both synergistic and RNA silencing suppression activities. Phytopathology 95, 894–901. 10.1094/PHYTO-95-0894 18944411

[B20] Guo B. Lin J. Ye K. (2011). Structure of the autocatalytic cysteine protease domain of potyvirus helper-component proteinase. J. Biol. Chem. 286, 21937–21943. 10.1074/jbc.M111.230706 21543324 PMC3122248

[B21] Haikonen T. Rajamäki M. L. Tian Y. P. Valkonen J. P. T. (2013). Mutation of a short variable region in HCpro Protein of *Potato virus A* affects interactions with a microtubule-associated protein and induces necrotic responses in tobacco. Mol. Plant-Microbe Interact. 26, 721–733. 10.1094/mpmi-01-13-0024-r 23514111

[B22] Hofgen R. Willmitzer L. (1988). Storage of competent cells for Agrobacterium transformation. Nucleic Acids Res. 16, 9877–9877. 10.1093/nar/16.20.9877 3186459 PMC338805

[B23] Huang T. Cui Y. Zhang X. (2014). Involvement of viral microRNA in the regulation of antiviral apoptosis in shrimp. J. Virol. 88, 2544–2554. 10.1128/jvi.03575-13 24352447 PMC3958074

[B24] Huang X. D. Fang L. Gu Q. S. Tian Y. P. Geng C. Li X. D. (2019). Cross protection against the watermelon strain of *Papaya ringspot virus* through modification of viral RNA silencing suppressor. Virus Res. 265, 166–171. 10.1016/j.virusres.2019.03.016 30910699

[B25] Ivanov K. I. Eskelin K. Bašić M. De S. Lõhmus A. Varjosalo M. (2016). Molecular insights into the function of the viral RNA silencing suppressor HCPro. Plant J. Cell Mol. Biol. 85, 30–45. 10.1111/tpj.13088 26611351

[B26] Janson B. F. Ellett C. W. (1963). A new Corn disease in Ohio. Plant Dis. Rep. 47, 1107–1108.

[B27] Ji S. X. Wang S. W. Wang J. Li X. D. Zhu T. S. Tian Y. P. (2018). Preparation and application of antiserum against watermelon mosaic virus coat protein expressed in *E. coli* . Acta Phytopathol. Sin. 48, 833–837. 10.13926/j.cnki.apps.000237

[B28] Jiang J. X. Zhou X. P. (2002). Maize dwarf mosaic disease in different regions of China is caused by *Sugarcane mosaic virus* . Arch. Virol. 147, 2437–2443. 10.1007/s00705-002-0890-7 12491109

[B29] Kamitani M. Nagano A. J. Honjo M. N. Kudoh H. (2016). RNA-Seq reveals virus-virus and virus-plant interactions in nature. FEMS Microbiol. Ecol. 92, fiw176. 10.1093/femsec/fw176 PMC5854034 27549115

[B30] Karyeija R. F. Kreuze J. F. Gibson R. W. Valkonen J. P. (2000). Synergistic interactions of a potyvirus and a phloem-limited crinivirus in sweet potato plants. Virology 269, 26–36. 10.1006/viro.1999.0169 10725195

[B31] Kasschau K. D. Carrington J. C. (1998). A counter defensive strategy of plant viruses: suppression of posttranscriptional gene silencing. Cell 95, 461–470. 10.1016/S0092-8674(00)81614-1 9827799

[B32] Kimalov B. Gal-On A. Stav R. Belausov E. Arazi T. (2004). Maintenance of coat protein N-terminal net charge and not primary sequence is essential for zucchini yellow mosaic virus systemic infectivity. J. Gen. Virol. 85, 3421–3430. 10.1099/vir.0.80417-0 15483260

[B33] Krstic B. Ford R. E. Shukla D. D. Tosic M. (1995). Cross-protection studies between strains of sugarcane mosaic, maize dwarf mosaic, Johnsongrass mosaic, and sorghum mosaic potyviruses. Plant Dis. 79, 135–138. 10.1094/PD-79-0135

[B34] Kung Y. J. Lin P. C. Yeh S. D. Hong S. F. Chua N. H. Liu L. Y. (2014). Genetic analyses of the FRNK motif function of *Turnip mosaic virus* uncover multiple and potentially interactive pathways of cross-protection. Mol. Plant-Microbe Interact. 27, 944–955. 10.1094/mpmi-04-14-0116-r 24804808

[B35] Kunkel L. (1955). Cross protection between strains of yellows-type viruses. Adv. Virus Res. 3, 251–273. 10.1016/S0065-3527(08)60638-7 13354520

[B36] Lan Y. F. Liu J. L. Gao R. Wang H. Y. Zhu T. S. Zhu X. P. (2007). Expression of tobacco vein banding mosaic virus coat protein in *E. coli* and preparation of antiserum. Acta Phytopathol. Sin. 37, 462–466. 10.13926/j.cnki.apps.2007.05.014

[B37] Lin S. S. Wu H. W. Jan F. J. Hou R. F. Yeh S. D. (2007). Modifications of the helper component-protease of *Zucchini yellow mosaic virus* for generation of attenuated mutants for cross protection against severe infection. Phytopathology 97, 287–296. 10.1094/phyto-97-3-0287 18943647

[B38] Mahuku G. Lockhart B. E. Wanjala B. Jones M. W. Kimunye J. N. Stewart L. R. (2015). Maize lethal necrosis (MLN), an emerging threat to maize-based food security in sub-Saharan Africa. Phytopathology 105, 956–965. 10.1094/phyto-12-14-0367-fi 25822185

[B39] Moradi Z. Nazifi E. Mehrvar M. (2017). Occurrence and evolutionary analysis of coat protein gene sequences of Iranian isolates of *Sugarcane mosaic virus* . Plant Pathol. J. 33, 296–306. 10.5423/PPJ.OA.10.2016.0219 28592948 PMC5461048

[B40] Nakahara K. S. Masuta C. Yamada S. Shimura H. Kashihara Y. Wada T. S. (2012). Tobacco calmodulin-like protein provides secondary defense by binding to and directing degradation of virus RNA silencing suppressors. Proc. Natl. Acad. Sci. U. S. A. 109, 10113–10118. 10.1073/pnas.1201628109 22665793 PMC3382489

[B41] Olspert A. Chung B. Y. W. Atkins J. F. Carr J. P. Firth A. E. (2015). Transcriptional slippage in the positive-sense RNA virus family *Potyviridae* . EMBO Rep. 16, 995–1004. 10.15252/embr.201540509 26113364 PMC4552492

[B42] Pacheco R. García-Marcos A. Barajas D. Martiáñez J. Tenllado F. (2012). PVX–potyvirus synergistic infections differentially alter microRNA accumulation in *Nicotiana benthamiana* . Virus Res. 165, 231–235. 10.1016/j.virusres.2012.02.012 22387565

[B43] Pechinger K. Chooi K. M. MacDiarmid R. M. Harper S. J. Ziebell H. (2019). A new era for mild strain cross-protection. Viruses 11, 670. 10.3390/v11070670 PMC6669575 31340444

[B44] Poque S. Wu H. W. Huang C. H. Cheng H. W. Hu W. C. Yang J. Y. (2017). Potyviral gene-silencing suppressor HCPro interacts with salicylic Acid (SA)-binding protein 3 to weaken SA-mediated defense responses. Mol. Plant-Microbe Interact. 31, 86–100. 10.1094/mpmi-06-17-0128-fi 29090655

[B45] Rast A. T. B. (1972). M II-16, an artificial symptomless mutant of tobacco mosaic virus for seedling inoculation of tomato crops. Neth. J. Plant Pathol. 78, 110–112. 10.1007/bf01980475

[B46] Rodamilans B. Valli A. Mingot A. San Leon D. Lopez-Moya J. J. Garcia J. A. (2018). An atypical RNA silencing suppression strategy provides a snapshot of the evolution of sweet potato-infecting potyviruses. Sci. Rep. 8, 15937. 10.1038/s41598-018-34358-y 30374036 PMC6206096

[B47] Roossinck M. J. (1997). Mechanisms of plant virus evolution. Annu. Rev. Phytopathol. 35, 191–209. 10.1146/annurev.phyto.35.1.191 15012521

[B48] Shiboleth Y. M. Haronsky E. Leibman D. Arazi T. Wassenegger M. Whitham S. A. (2007). The conserved FRNK box in HC-Pro, a plant viral suppressor of gene silencing, is required for small RNA binding and mediates symptom development. J. Virol. 81, 13135–13148. 10.1128/jvi.01031-07 17898058 PMC2169133

[B49] Sun L. Suzuki N. (2008). Intragenic rearrangements of a mycoreovirus induced by the multifunctional protein p29 encoded by the prototypic hypovirus CHV1-EP713. RNA 14, 2557–2571. 10.1261/rna.1125408 18945807 PMC2590959

[B50] Sun M. X. Kong Q. Yuan J. Niu P. Xie J. Jiang W. (2014). Screening suitable reference genes for normalization in reverse transcription quantitative real-time PCR analysis in Melon. PloS One 9, e87197. 10.1371/journal.pone.0087197 24475250 PMC3903635

[B51] Takeshita M. Shigemune N. Kikuhara K. Furuya N. Takanami Y. (2004). Spatial analysis for exclusive interactions between subgroups I and II of *Cucumber mosaic virus* in cowpea. Virology 328, 45–51. 10.1016/j.virol.2004.06.046 15380357

[B52] Tanzi M. Betti L. De Jager C. Canova A. (1987). Isolation of an attenuated virus mutant obtained from a TMV pepper strain after treatment with nitrous acid. Phytopathol. Mediterr. 25, 119–124.

[B53] Tomlinson J. Shepherd R. (1978). Studies on mutagenesis and cross-protection of cauliflower mosaic virus. Ann. Appl. Biol. 90, 223–231. 10.1111/j.1744-7348.1978.tb02630.x

[B54] Torres-Barceló C. Martín S. Daròs J. A. Elena S. F. (2008). From hypo-to hypersuppression: effect of amino acid substitutions on the RNA-silencing suppressor activity of the *Tobacco etch potyvirus* HC-Pro. Genetics 180, 1039–1049. 10.1534/genetics.108.091363 18780745 PMC2567354

[B55] Torres-Barceló C. Daròs J. A. Elena S. F. (2009). Compensatory molecular evolution of HC-Pro, an RNA-silencing suppressor from a plant RNA virus. Mol. Biol. Evol. 27, 543–551. 10.1093/molbev/msp272 19906792

[B56] Tuo D. Zhou P. Zhao G. Yan P. Tan D. Li X. (2020). A double mutation in the conserved motifs of the helper component protease of papaya leaf distortion mosaic virus for the generation of a cross-protective attenuated strain. Phytopathology 110, 187–193. 10.1094/PHYTO-09-19-0328-R 31516080

[B57] Untiveros M. Fuentes S. Salazar L. F. (2007). Synergistic interaction of sweet potato chlorotic stunt virus (Crinivirus) with Carla-, Cucumo-, Ipomo-, and potyviruses infecting sweet potato. Plant Dis. 91, 669–676. 10.1094/PDIS-91-6-0669 30780474

[B58] Valli A. Gallo A. Calvo M. de Jesus Perez J. Garcia J. A. (2014). A novel role of the potyviral helper component proteinase contributes to enhance the yield of viral particles. J. Virol. 88, 9808–9818. 10.1128/JVI.01010-14 24942578 PMC4136352

[B59] Valli A. A. Gallo A. Rodamilans B. Lopez-Moya J. J. Garcia J. A. (2018). The HCPro from the *Potyviridae* family: an enviable multitasking Helper Component that every virus would like to have. Mol. Plant Pathol. 19, 744–763. 10.1111/mpp.12553 28371183 PMC6638112

[B60] Wang Y. Cong Q. Q. Lan Y. F. Geng C. Li X. D. Liang Y. C. (2014). Development of new potato virus X-based vectors for gene over-expression and gene silencing assay. Virus Res. 191, 62–69. 10.1016/j.virusres.2014.07.018 25076104

[B61] Wu L. Zu X. Wang S. Chen Y. (2012). *Sugarcane mosaic virus* – Long history but still a threat to industry. Crop Prot. 42, 74–78. 10.1016/j.cropro.2012.07.005

[B62] Xia Z. Zhao Z. Chen L. Li M. Zhou T. Deng C. (2016). Synergistic infection of two viruses MCMV and SCMV increases the accumulations of both MCMV and MCMV-derived siRNAs in maize. Sci. Rep. 6, 1–12. 10.1038/srep20520 26864602 PMC4808907

[B63] Xie X. Chen W. Fu Q. Zhang P. An T. Cui A. (2016). Molecular variability and distribution of *Sugarcane mosaic virus* in Shanxi, China. PloS One 11, e0151549. 10.1371/journal.pone.0151549 26987060 PMC4795778

[B64] Xu X. J. Zhang J. W. Xu D. K. Yin F. W. Tian Y. P. Li X. D. (2018). Prokaryotic expression and antiserum preparation of sugarcane mosaic virus coat protein. Shandong Agric. Sci. 50, 106–109. 10.14083/j.issn.1001-4942.2018.08.022

[B65] Xu X. J. Yu W. P. Yang G. L. Han S. L. He M. J. Yang X. (2019). Prokaryotic expression and antiserum preparation of helper component– proteinase of *Sugarcane mosaic virus* . Shandong Agric. Sci. 51, 87–91. 10.14083/j.issn.1001-4942.2019.03.018

[B66] Yambao M. L. Yagihashi H. Sekiguchi H. Sekiguchi T. Sasaki T. Sato M. (2008). Point mutations in helper component protease of clover yellow vein virus are associated with the attenuation of RNA-silencing suppression activity and symptom expression in broad bean. Arch. Virol. 153, 105–115. 10.1007/s00705-007-1073-3 17955160

[B67] Yan Z. Y. Cheng D. J. Liu J. Tian Y. P. Zhang S. B. Li X. D. (2016). First Report of *Sugarcane mosaic virus* group IV Isolates from the Corn Production Fields in China. Plant Dis. 100, 1508. 10.1094/PDIS-11-15-1373-PDN

[B68] Yang S. Ravelonandro M. (2002). Molecular studies of the synergistic interactions between plum pox virus HC-Pro protein and potato virus X. Arch. Virol. 147, 2301–2312. 10.1007/s00705-002-0892-5 12491099

[B69] You B. J. Chiang C. H. Chen L. F. Su W. C. Yeh S. D. (2005). Engineered mild strains of *Papaya ringspot virus* for broader cross protection in cucurbits. Phytopathology 95, 533–540. 10.1094/PHYTO-95-0533 18943319

[B70] Zhang C. Wu Z. Li Y. Wu J. (2015). Biogenesis, function, and applications of virus-derived small RNAs in plants. Front. Microbiol. 6, 1237. 10.3389/fmicb.2015.01237 26617580 PMC4637412

[B71] Zhang X. F. Guo J. Zhang X. Meulia T. Paul P. Madden L. V. (2015). Random Plant Viral Variants Attain Temporal Advantages During Systemic Infections and in Turn Resist other Variants of the Same Virus. Sci. Rep. 5, 15346. 10.1038/srep15346 26481091 PMC4612314

[B72] Zhou C. J. Zhang X. Y. Liu S. Y. Wang Y. Li D. W. Yu J. L. (2017). Synergistic infection of BrYV and PEMV 2 increases the accumulations of both BrYV and BrYV-derived siRNAs in *Nicotiana benthamiana* . Sci. Rep. 7, 45132. 10.1038/srep45132 28345652 PMC5366869

[B73] Ziebell H. Carr J. P. (2009). Effects of dicer-like endoribonucleases 2 and 4 on infection of *Arabidopsis thaliana* by cucumber mosaic virus and a mutant virus lacking the 2b counter-defence protein gene. J. Gen. Virol. 90, 2288–2292. 10.1099/vir.0.012070-0 19474248

[B74] Ziebell H. MacDiarmid R. (2017). Prospects for engineering and improvement of cross-protective virus strains. Curr. Opin. Virol. 26, 8–14. 10.1016/j.coviro.2017.06.010 28743041

