# Supplementary material for: A Spontaneous Complementary Mutation Restores the RNA Silencing Suppression Activity of HC-Pro and the Virulence of Sugarcane Mosaic Virus
Source: Front Plant Sci. 2020 Aug 21;11:1279. doi: 10.3389/fpls.2020.01279 (PMC7472499; doi:10.3389/fpls.2020.01279)
Supplement: Supplementary file 1 [file DataSheet_1.docx]

**FIGURE S1 | Alignment of the FRNK motif in HC-Pros of six potyviruses.** The red triangles pointed to the amino acids (numbered as in SCMV HC-Pro) for mutation.

**FIGURE S2 | The HC-Pro coding sequences of the SCMV progeny.** The maize plants were challenge inoculated with intervals of 5, 7, and 10 days. The upper non-inoculated (systemic) maize leaves were collected at ten days post challenge inoculation. The codons of the amino acid at position 184 in SCMV HC-Pro were underlined in red.

**FIGURE S3 | Alignment of partial HC-Pro amino acid sequences** **of SCMV, WMV, and TVBMV.** The corresponding amino acids for G440 in SCMV HC-Pro (N437 in WMV HC-Pro and S438 in TVBMV HC-Pro) were indicated in the red box.

**FIGURE S4 | The stability of SCMV HC-Pro and its mutants in *E. coli*.** Purified maltose binding protein (MBP)-tagged HC-Pro proteins were separated and stained by Coomassie brilliant blue. M, marker.

**FIGURE S5 | Alignment of HC-Pro amino acid sequences from fifteen potyviruses.** The corresponding amino acids for G440 in SCMV HC-Pro were indicated in the red box.

**FIGURE S6 | The 3D structures of the cysteine protease domain of SCMV** **HC-Pro and its mutants (from aa 338-460).** HC-G440R, HC-G440K and HC-G440H: SCMV HC-Pro mutants with additional mutation of G440 to R, K, and H, respectively. HC-WT, wild type HC-Pro.

**FIGURE S7 | The GFP accumulation levels of SCMV-GFP at ten days post challenge inoculation.** CBB, Coomassie brilliant blue. The experiments were repeated three times independently.
