# Supplementary material for: A Spontaneous Complementary Mutation Restores the RNA Silencing Suppression Activity of HC-Pro and the Virulence of Sugarcane Mosaic Virus
Source: Front Plant Sci. 2020 Aug 21;11:1279. doi: 10.3389/fpls.2020.01279 (PMC7472499; doi:10.3389/fpls.2020.01279)
Supplement: Supplementary file 2 [file Table_1.docx]

**Supplemental Table 1 The name and sequence of the primers used in this study**

| Purpose | Primer name | Primer sequence (5'-3') |
| --- | --- | --- |
| SCMV mutants | SCMV-HC_R184I_-F | GGATTCATTTattAACAAGATCTCACCAAAGAGCACGATC |
|  | SCMV-HC_R184I_-R | GATCTTGTTaatAAATGAATCCAATGTGTCCGTTTTAAGTGATTC |
|  | SCMV-HC_R184K_-F | ATTCATTTaaaAACAAGATCTCACCAAAGAGCACGATCAATG |
|  | SCMV-HC_R184K_-R | GAGATCTTGTTtttAAATGAATCCAATGTGTCCGTTTTAAGTG |
|  | SCMV-HC_K186A_-F | GAAACgccATCTCACCAAAGAGCACGATCAATGC |
|  | SCMV-HC_K186A_-R | GTGAGATggcGTTTCGAAATGAATCCAATGTGTCC |
|  | SCMV-HC_K186R_-F | GAAAccgCATCTCACCAAAGAGCACGATCAATGC |
|  | SCMV-HC_K186R_-R | GTGAGATGcggTTTCGAAATGAATCCAATGTGTCC |
|  | SCMV-HC_G440R_-F | GTACCGTGagaCAACTGATAAAATTCCAGTACGAATCATTAGAAAG |
|  | SCMV-HC_G440R_-R | ATCAGTTGtctCACGGTACTTGCCTTTAGAATGTGGAATC |
|  | SCMV-HC_G440H_-F | GTACCGTGcatCAACTGATAAAATTCCAGTACGAATCATTAGAAAG |
|  | SCMV-HC_G440H_-R | ATCAGTTGatgCACGGTACTTGCCTTTAGAATGTGGAATC |
|  | SCMV-HC_G440K_-F | GTACCGTGaagCAACTGATAAAATTCCAGTACGAATCATTAGAAAG |
|  | SCMV-HC_G440K_-R | ATCAGTTGcttCACGGTACTTGCCTTTAGAATGTGGAATC |
| WMV mutants | WMV-HC_R181I_-F | AAGTATTCattAATAAGCGATCCTCCAAAGCATTGCTCAACCCCAG |
|  | WMV-HC_R181I_-R | GATCGCTTATTaatGAATACTTTAAGTGCGTCCTCATCAGTCAAG |
|  | WMV-HC_N437R_-F | GGCACAGTAaggCAACTCATTCAATTCGCATCAAATGATTTGCAC |
|  | WMV-HC_N437R_-R | GAGTTGcctTACTGTGCCAGCTTTCAAAACATGATATCCGACCGTC |
| TVBMV mutants | TVBMV-HC_R182I_-F | CTCGCAACGTTCataAATAAAGTTTCGGCAAAAGCTCACA |
|  | TVBMV-HC_R182I_-R | TTATTtatGAACGTTGCGAGCGATCCCTTCTTAATATTGTCAGTCC |
|  | TVBMV-HC_S438R_-F | CAAACACAGTTagaCAACTAGAAAAGTTTGCAAGTGATACACTTGAG |
|  | TVBMV-HC_S438R_-R | CTAGTTGtctAACTGTGTTTGCCTTCAGAATGTGATATCCTGTGGT |
| Real-time RT-PCR | SCMV-CP-qRT-F | GGCGAGACTCAGGAGAATACA |
|  | SCMV-CP-qRT-R | ACACGCTACACCAGAAGACACT |
|  | *ZmUbi*-qRT-F | GGAAAAACCATAACCCTGGA |
|  | *ZmUbi*-qRT-R | ATATGGAGAGAGGGCACCAG |
|  | TVBMV-CP-qRT-F | AACGCCAACCAGAGCAAGA |
|  | TVBMV-CP-qRT-R | ACATTTCCATCCAGTCCAAACA |
|  | *actin*-qRT-F | CTGATGAAGATACTCACAGAAAGAG |
|  | *actin*-qRT-R | CAGGATACGGGGAGCTAATG |
|  | WMV-CP-qRT-F | GTGGCAAAGGTGATAAGC |
|  | WMV-CP-qRT-R | GAGCAAATGGTCTAAACTGA |
|  | *EF1α*-qRT-F | CCACGAGTCTCTCCCAGAAG |
|  | *EF1α*-qRT-R | CACGCTTGAGATCCTTGACA |
|  | GFP-qRT-F | GTGGAGAGGGTGAAGGTGAT |
|  | GFP-qRT-R | CGGATAACGGGAAAAGCATTGA |
| Transient expression | pBin-SCMV-HC-F | CGGGATCCATGGCTGATCCACAAGCGAATAG |
|  | pBin-SCMV-HC-R | GGAGCTCTTATCCCACTATATATTCACGCATCTC |
|  | pBin-WMV-HC-F | CGGGATCCATGTCTCACACTCCAGAAGTTCAATTTTTCC |
|  | pBin-WMV-HC-R | GGAGCTCTTAGCCAACCCTGTAAAATTTCATTTCAC |
|  | pBin-TVBMV-HC-F | CGGGATCCATGTCAGCGGCAGAGCAATTTTGG |
|  | pBin-TVBMV-HC-R | GGAGCTCTTAGCCAACTCTATAATGCTTCATTTC |

The underlined letters represented restriction sites; the mutation sites were in lower case.
