# Supplementary figures and images for: A Spontaneous Complementary Mutation Restores the RNA Silencing Suppression Activity of HC-Pro and the Virulence of Sugarcane Mosaic Virus
Source: Front Plant Sci. 2020 Aug 21;11:1279. doi: 10.3389/fpls.2020.01279 (PMC7472499; doi:10.3389/fpls.2020.01279)

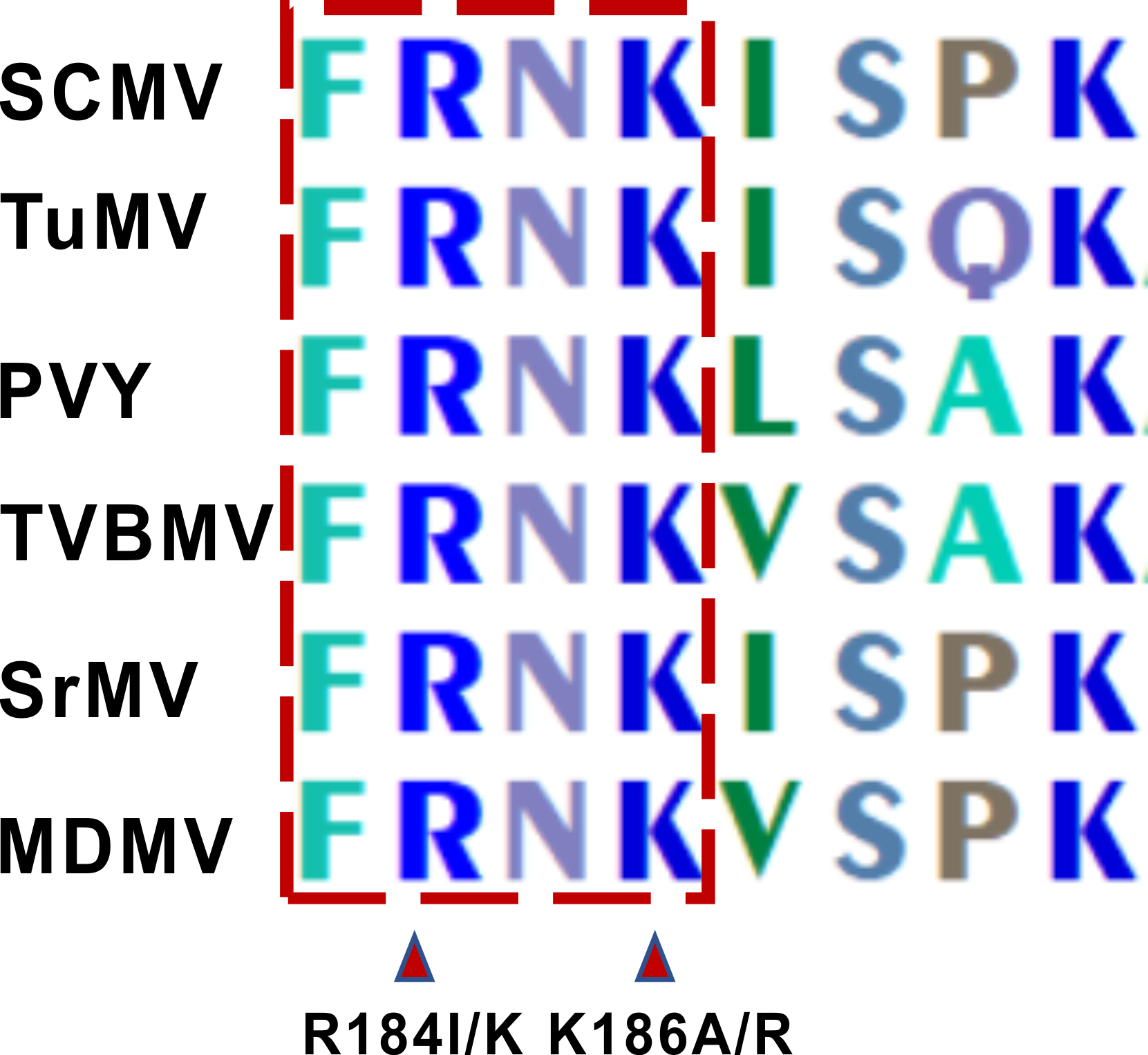

Supplement: Figure S1 — Alignment of the FRNK motif in HC-Pros of six potyviruses. The red triangles pointed to the amino acids (numbered as in SCMV HC-Pro) for mutation. [file Image_1.tif]

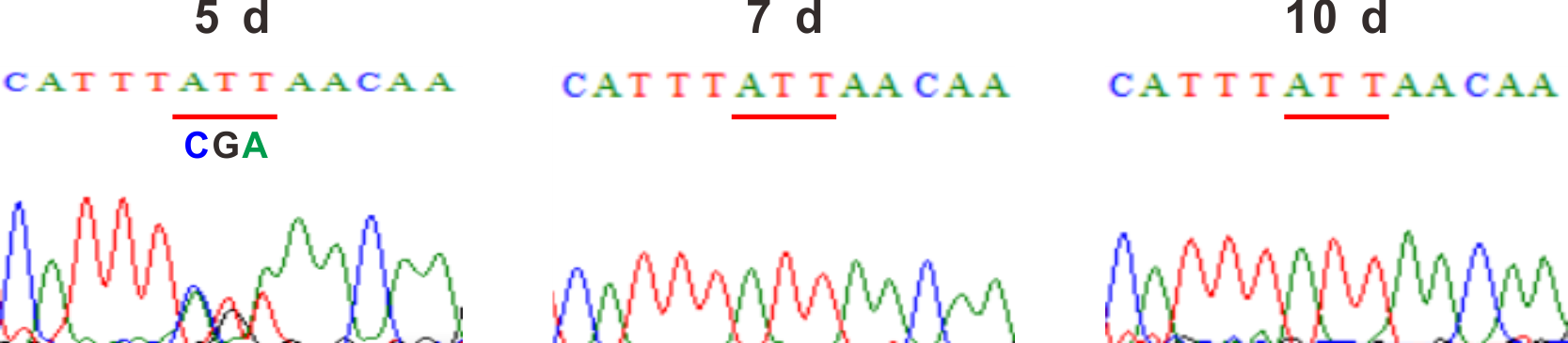

Supplement: Figure S2 — The HC-Pro coding sequences of the SCMV progeny. The maize plants were challenge inoculated with intervals of 5, 7, and 10 days. The upper non-inoculated (systemic) maize leaves were collected at ten days post challenge inoculation. The codons of the amino acid at position 184 in SCMVHC-Pro were underlined in red. [file Image_2.tif]

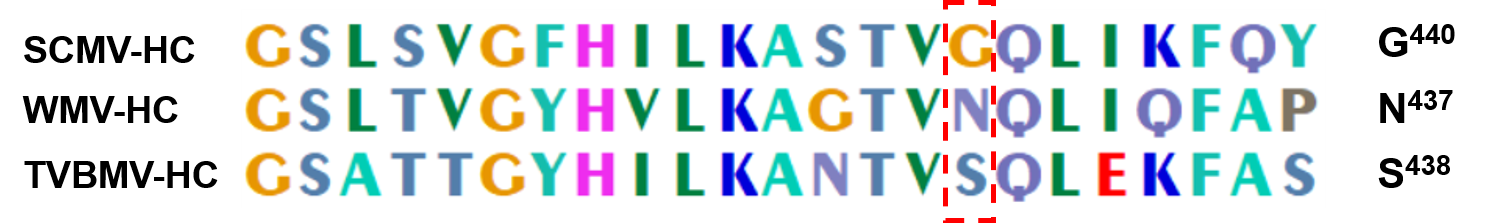

Supplement: Figure S3 — Alignment of partial HC-Pro amino acid sequences of SCMV, WMV, and TVBMV. The corresponding amino acids for G440 in SCMV HC-Pro (N437 in WMV HC-Pro and S438 in TVBMVHC-Pro) were indicated in the red box. [file Image_3.tif]

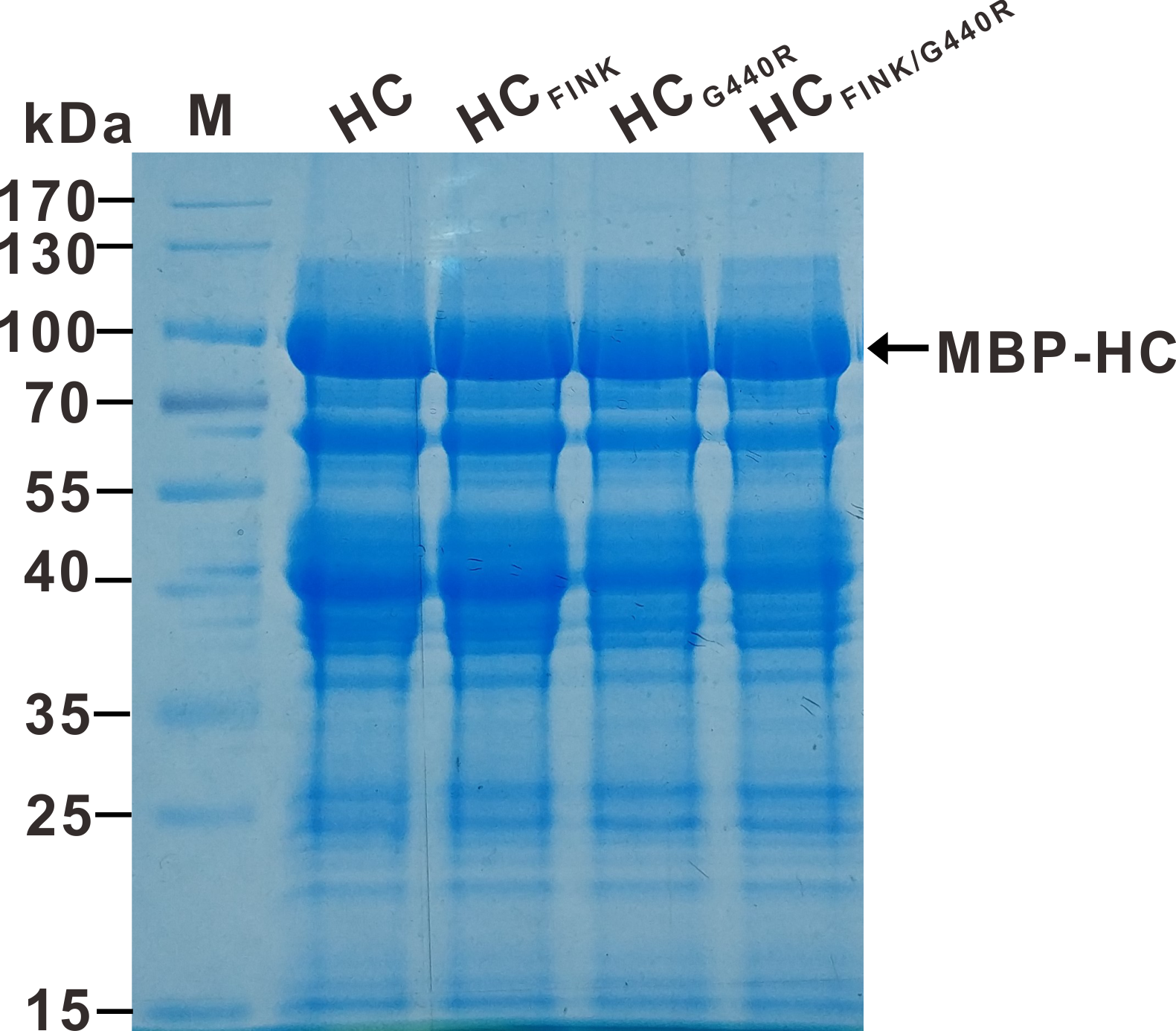

Supplement: Figure S4 — The stability of SCMV HC-Pro and its mutants in E. coli. Purified maltose binding protein (MBP)-tagged HC-Pro proteins were separated and stained by Coomassie brilliant blue. M, marker. [file Image_4.tif]

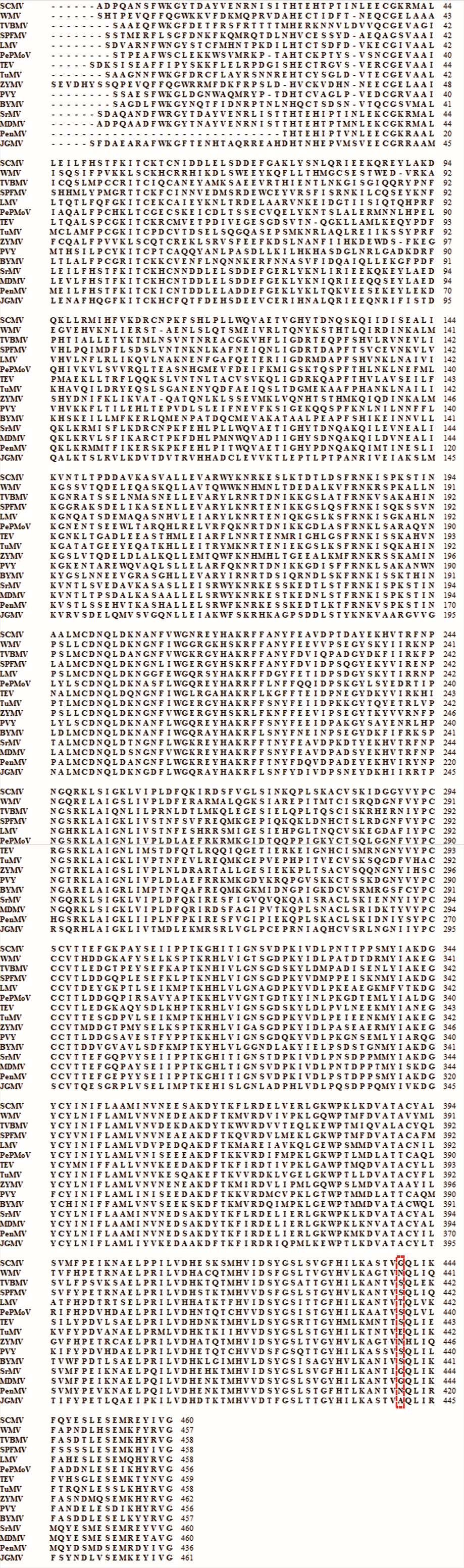

Supplement: Figure S5 — Alignment of HC-Pro amino acid sequences from fifteen potyviruses. The corresponding amino acids for G440 in SCMV HC-Pro were indicated in the red box. [file Image_5.tif]

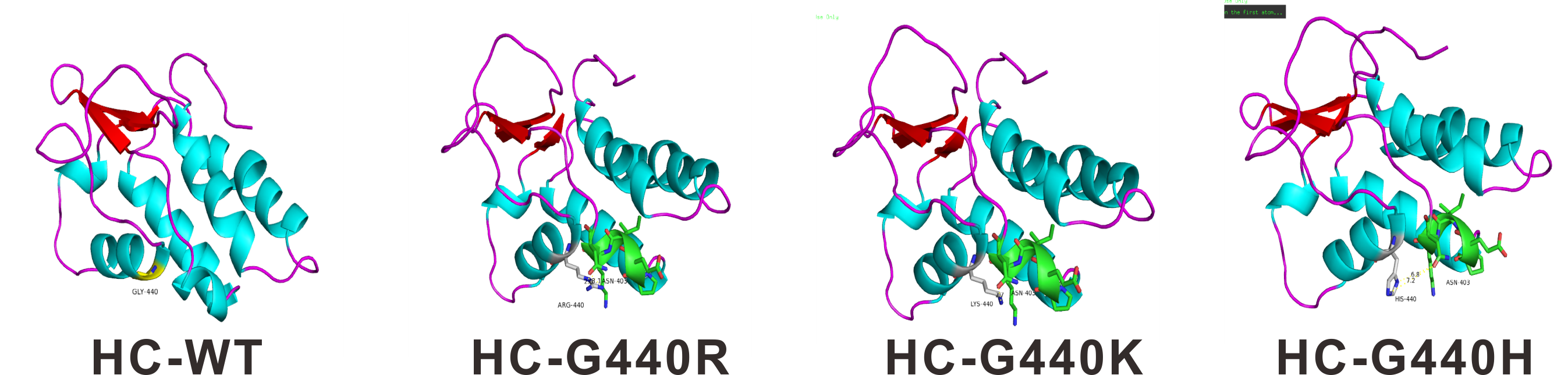

Supplement: Figure S6 — The 3D structures of the cysteine protease domain of SCMVHC-Pro and its mutants(from aa 338-460). HC-G440R, HC-G440K and HC-G440H: SCMV HC-Pro mutants with additional mutation of G440 to R, K, and H, respectively. HC-WT, wild type HC-Pro. [file Image_6.tif]

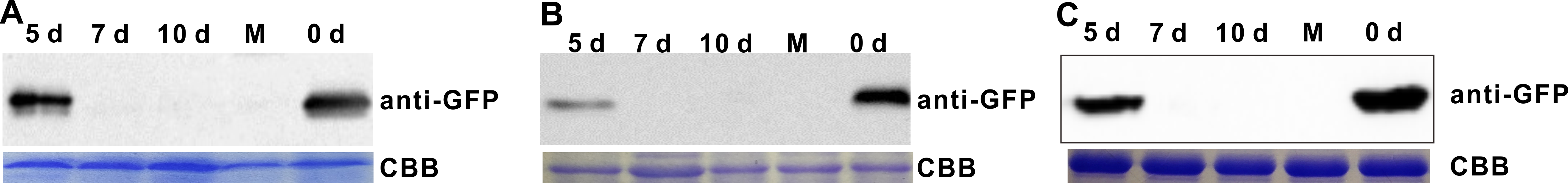

Supplement: Figure S7 — The GFP accumulation levels of SCMV-GFP at ten days post challenge inoculation.CBB, Coomassie brilliant blue. The experiments were repeated three times independently. [file Image_7.tif]
